# Deep excitation afterglow luminescent probes for biomedical applications

**DOI:** 10.1039/d5sc09312k

**Published:** 2026-03-10

**Authors:** Yiqian Hao, Yuxia Liu, Xi Liu, Siyue Ma, Chao Wang, Qing Miao, Linlin Wang, Pu Chen, Dongliang Su, Jonathan L. Sessler, Bo Tang, Tony D. James, Guang Chen

**Affiliations:** a Shaanxi Key Laboratory of Chemical Additives for Industry, College of Chemistry and Chemical Engineering, Shaanxi University of Science & Technology Xi'an 710021 China chenandguang@163.com liuyuxia2008@163.com; b Department of Chemistry, University of Bath Bath BA2 7AY UK t.d.james@bath.ac.uk xizai901226@163.com; c Department of Psychiatry, Shandong Mental Health Center, Shandong University Jinan 250014 China; d Ningbo Key Laboratory of All-Solid-State Battery, Eastern Institute for Advanced Study, Eastern Institute of Technology Ningbo 315200 China puchen@eitech.edu.cn; e Department of Chemistry, The University of Texas at Austin Texas 78712-1224 USA sessler@cm.utexas.edu; f College of Chemistry, Chemical Engineering and Materials Science, Shandong Normal University Jinan 250014 China tangb@sdnu.edu.cn; g Yimingtai Biotechnology Co., Ltd No. 507 Binhe Avenue, Development Zone Taian 271200 China; h School of Chemistry and Chemical Engineering, Henan Normal University Xinxiang 453007 China

## Abstract

In addition to the high sensitivity, excellent spatio-temporal resolution and powerful real-time imaging capabilities, biomedical applications impose high demands on imaging techniques. Unfortunately, conventional imaging relies on the real-time excitation and suffers from limited tissue penetration. In contrast, afterglow imaging can provide continuous and deep luminescence once the probe is excited by NIR-light, X-ray or ultrasound. As such, it can effectively avoid autofluorescence and improve the imaging sensitivity and signal-to-background ratio. Moreover, X-ray-activated afterglow probes benefit from enhanced depth of penetration, thereby allowing more effective imaging of deep-seated lesions. Such advantages have attracted the interest of researchers, which should speed up the translation of biomedical afterglow research for clinical applications. With this perspective, we provided a comparative and analytical summary of the latest advances while highlighting the most promising afterglow probes. This perspective also outlines forward-looking strategies for molecular design, working mechanisms, and clinical prospects. Moreover, future challenges and research directions are discussed. As such this perspective describes how to formulate the most promising chemical strategies through mechanistic understanding, molecular design, and functional integration, thereby maximizing the successful development of clinical probes for visualization in humans.

## Abstract 2

Afterglow imaging provides continuous deep luminescence when excited by NIR-light, X-ray or ultrasound. Avoiding autofluorescence, improving imaging sensitivity and signal-to-background ratio.

## Graphical abstract

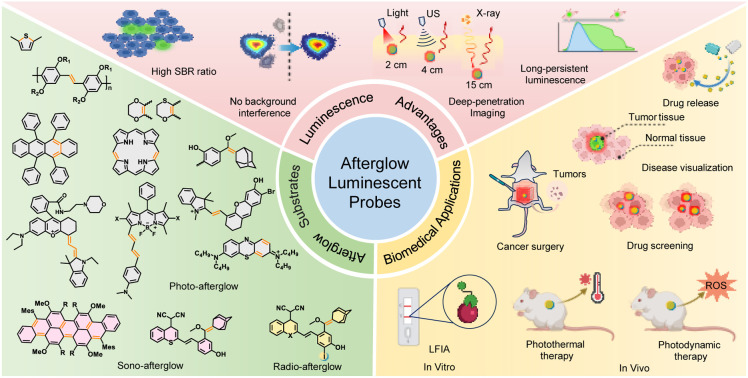

## 1. Introduction

**Fig. 1:**
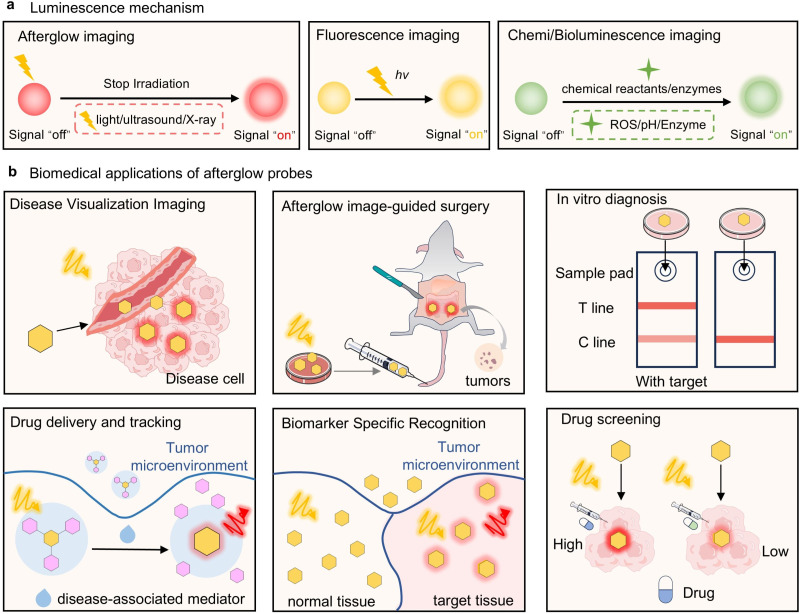
(a) Optical imaging modalities. (b) Biomedical applications of molecular afterglow imaging.

Optical imaging is a direct and powerful tool for biomedical research.^1–7^ It can reveal dynamic and detailed changes in pathophysiological processes, with spatio-temporal resolution and imaging sensitivity.^8–11^ Furthermore, optical imaging has the advantage of non-invasiveness and real-time visualization. These characteristics make optical imaging of significant interest in the context of biomedical research and clinical translation.^12–15^ Unfortunately, fluorescence imaging often suffers significant background noise due to tissue autofluorescence under real-time light excitation (Fig. 1).^16–20^ Chemiluminescence/bioluminescence (Chemi/Bioluminescence) imaging does not require continuous real-time excitation, thus it can avoid such background noise.^21–24^ However, these approaches often require the addition external active substances or endogenous enzymes to continuously trigger the luminescence. These limitations have hindered their widespread use *in vivo*. In contrast, the unique feature of afterglow luminescence is that the probe can continue to emit light after excitation, without the need for continuous excitation or triggering.^25–29^ This process effectively avoids interference from tissue fluorescence and improves the imaging sensitivity and signal-to-background ratio (SBR). Moreover, once combined with ultrasound or X-rays, the penetration depth can be enhanced, which it is hoped will promote the translation of afterglow imaging techniques into clinical practice.^30–33^

These superior imaging properties stem from its unique “energy conversion-chemical energy storage-slow light emission” mechanism. Simply put, organic afterglow luminescence is essentially a multi-step process involving external energy capture, chemical energy storage, and slow photon release: first, the probe captures external excitation energy (such as light, ultrasound, or X-rays) and then converts it into storable chemical energy. This process induces the generation of reactive oxygen species (ROS), primarily singlet oxygen (^1^O_2_). Specifically, light excitation directly triggers photosensitizers to generate ROS, ultrasound converts mechanical waves into chemical energy to induce ROS production, and X-rays absorb high-energy photons *via* the photoelectric effect, converting them into chemical energy and inducing ROS generation. Second, ROS undergo oxidation reactions with substrates within the probe (*e.g.*, conjugated polymers, small-molecule frameworks), forming high-energy intermediates (*e.g.*, dioxetanes, epoxides) that store energy in chemical form. Finally, after excitation ceases, these unstable high-energy intermediates slowly degrade and undergo radiative transitions, continuously releasing photons to generate afterglow luminescence removing the need for real-time excitation. This mechanism enables organic afterglow luminescence to function independently of continuous external illumination without requiring additional active substances or enzymes. Consequently, the luminescence signal remains stable and is less susceptible to interference from the biological environment. It is precisely this characteristic that grants afterglow imaging exceptionally high sensitivity and SBR, establishing a crucial foundation for deep tissue imaging.

**Fig. 2:**
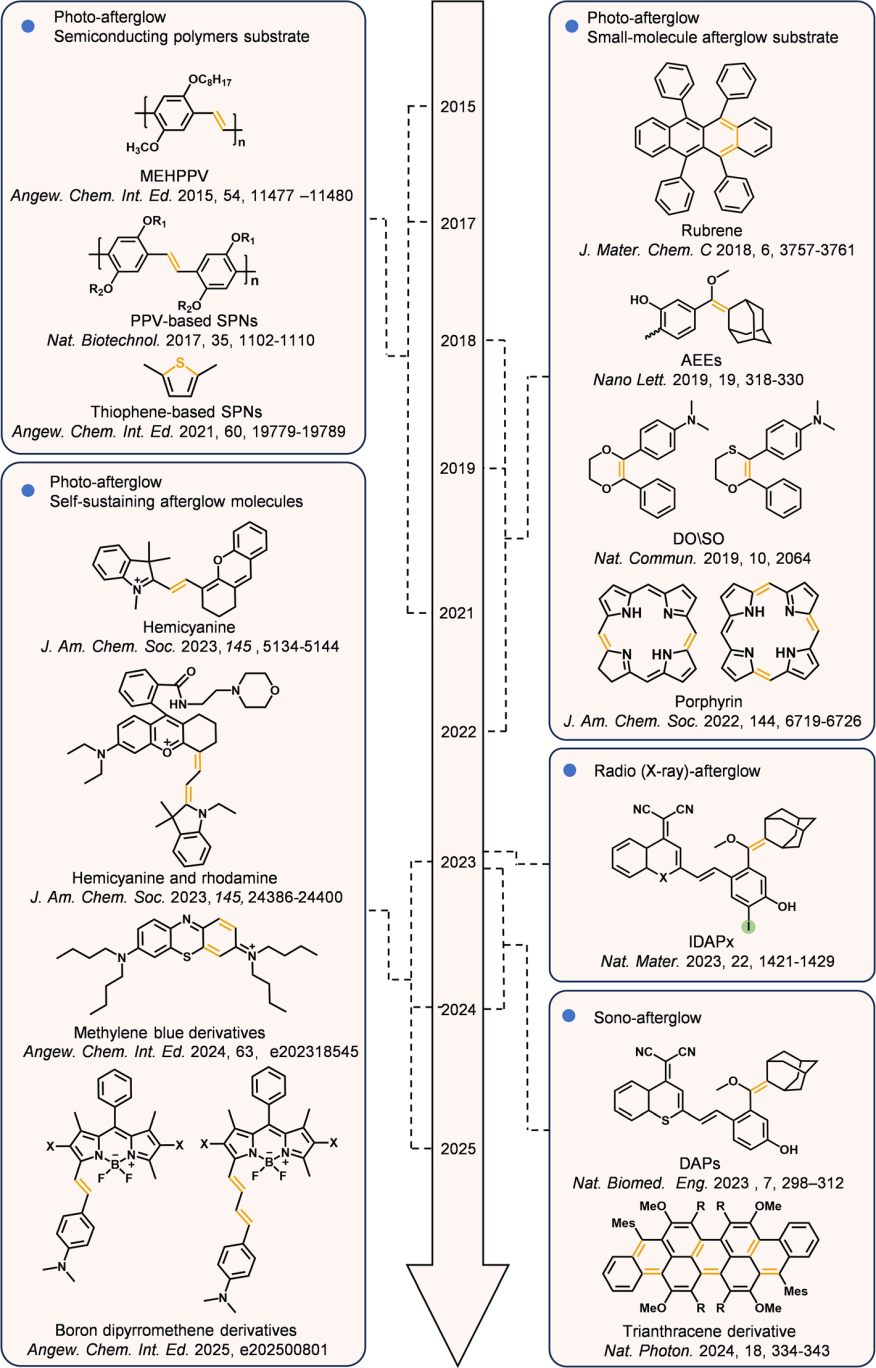
Organic afterglow luminescent imaging probes for biomedical diagnosis applications. The figure presents the chemical structures of representative afterglow substrates using different excitation types (photo-afterglow, radio/X-ray-afterglow, sono-afterglow). It also lists the first representative reports of various substrates (not all reports), while also showing their subsequent development.

In recent years, the development of afterglow imaging probes has progressed with remarkable speed. Rao *et al.* reported the first MEH-PPV (poly(2-methoxy-5-(2-ethylhexyloxy)-1,4-styryl))-based organic afterglow imaging probe, pioneering the field of afterglow imaging.^34^ Pu and colleagues further observed that semiconducting polymer nanoparticles (SPNs) based on polyphenylene vinylene (PPV) generate afterglow luminescence after light excitation.^35^ Through systematic research, the internal mechanism of afterglow luminescence has been elucidated: Briefly, it involves a process wherein dioxetane intermediates induced by ^1^O_2_ slowly degrade to release photons. On this basis, researchers have successfully constructed SPNs with better performance by modifying the molecular structure of PPV, such as introducing PEG chains and incorporating photosensitizers.^36–41^ Subsequently, researchers have expanded the scope of this approach to include other conjugated polymer systems. This led to the discovery that thiophene-based SPNs are also suitable for *in vivo* afterglow luminescence imaging, providing a new direction for the design of organic afterglow probes.^42,43^ Moving beyond SPNs, Ding *et al.* developed organic afterglow imaging probes based on a small-molecule afterglow substrates: Exploiting chemiluminescent adamantylidene enol ethers (AEEs) as the afterglow substrate.^44^ In this case a photosensitizer was introduced to generate ^1^O_2_, triggering the oxidation of the AEEs to form unstable high-energy intermediates. These intermediates release energy through chemiexcitation decomposition, ultimately achieving afterglow luminescence. Following this strategy, other organic afterglow luminescent probes using small molecules as substrates have been successively reported, including 1,4-oxathiin (*N*,*N*-dimethyl-4-(2-phenyl-5,6-dihydro-1,4-oxathiolan-3-yl) aniline, SO)^45^ and its derivatives,^46,47^ 1,4-dioxin (*N*,*N*-dimethyl-4-(3-phenyl-5,6-dihydro-1,4-dioxin-2-yl) aniline, DO),^45,48,49^ cypridina luciferin analogs (CLA),^50^ and rubrene.^51^ These probes provide more diverse options for biomedical applications in different scenarios. In 2022, Ding's team and Miao's group separately reported organic afterglow imaging probes based on small-molecule porphyrin derivatives.^52,53^ These probes exhibit excellent near-infrared afterglow luminescence performance, enabling accurate imaging of intraperitoneal micro-metastatic 4T1 tumors in mice and assisting in tumor resection during surgical navigation. This provides a new approach for the application of afterglow imaging in deep-tissue imaging and early-stage tumor detection. Against this backdrop, the field of afterglow imaging has experienced rapid development, with multiple novel probe systems emerging in succession: hemicyanine-based and self-sustaining afterglow probes have proved effective, with several enabling highly sensitive afterglow imaging through structural tunability.^54–58^ Likewise TPP-DO, a 1,4-dioxin–tetraphenylporphyrin conjugate^49^ and tri-anthracene derivatives (TAD)^59^ have shown significant potential for enhancing the brightness and prolonging the duration of persistent luminescence. While imaging probes based on photoexcited afterglow luminescence exhibit excellent performance, their energy transfer efficiency for deep tissues is still limited. To address this problem, Pu and his team developed an ultrasound-activated afterglow probe (sono-afterglow).^30,60^ The probe converts mechanical waves into chemical energy under ultrasonic excitation to produce afterglow luminescence. This strategy allows for a penetration depth of more than 4 cm into solid tissues. While the team of Tan constructed trianthracene derivative-based nanoparticles (TD NPs) with high luminescence intensity and longer afterglow duration *via* the synergy of the piezoelectric effect and chemiluminescence.^31^ In addition, Pu's team developed an X-ray (radio)-activated afterglow probe (radio-afterglow) that converts the absorbed X-ray energy into chemical energy,^32,33^ enabling even deeper tissue diagnosis and treatment (up to 15 cm). Collectively, these methods provide novel solutions to the longstanding challenge of improving the diagnosis of deep lesions (Fig. 2 and Table 1).

**Table 1:** Properties of representative afterglow luminescent probes for biomedical applications^a^

Material	System	*λ*_em_ (nm)	Brightness (p/s/sr/cm^2^)	*t*_1/2_ (min)	Imaging SBR	PD (cm)	Applications	Ref.
**Poly(*p*-phenylenevinylene) and its analogs**								
SPN-NCBS	MEHPPV/NCBS/PEG-*b*-PPG-*b*-PEG	780	2.2 × 10^7^	6.6	419 (s.c./12.5 µg)	4	Tumor and lymph node imaging	35
SPN2.5	PPV-TPP/PEG-*b*-PPG-*b*-PEG	720	1.5 × 10^6^	5	27.6 (i.v./80 µg)	—	Peritoneal metastatic tumor imaging	37
SPPVN	PPV-PEGL	775	1.1 × 10^5^	4.8	4170 (s.c./6.5 µg)	—	Peritoneal metastatic tumor imaging	36
ASPNC	ASP/Apt_CD63_	680	—	—	—	—	Multiplex differentiation of cancer exosomes	65
APPN	PBBTOT/NCBS/PPV-PEG-cRGD	780	—	2.1	33.8 (i.v./80 µg)	—	Imaging-guided post-BCS adjuvant NIR-II PTT	38
F1^2+^-ANP-Gal	F1^2+^/MEH-PPV/NIR-775/DSPE-PEG	780	—	6.6	124.5 (i.v./20 µg)	4	Orthotopic liver tumor imaging	66
ALCNs	AIN(MEH-PPV/C_18_-PEG_2000_-DA)/AIN(NCBS-PS-*b*-PAA)	780	4.5 × 10^6^	2	220 (i.v./4 mg kg^−1^)	—	Tumor imaging	39

**Thiophene-based SPs**								
PFODBT@CPPO	PFODBT/CPPO/F127	700	7.0 × 10^6^	2.2	48 (i.t./60 µg)	—	Monitoring ROS in photodynamic therapy	42
SPN(NIR-3)	NIR-3/DSPE-PEG_2000_	800	9.0 × 10^5^	0.86	155 (i.t./50 µg)	2	Monitoring ROS in photodynamic therapy	43

**Adamantylidene enol ethers**								
APtN	NCBS/PEG-AE-5-DFUR (AGL-1)	550	1.35 × 10^6^	16	—	—	Drug tracking	62
PA-AGL NPs	AGL-4/TPE-TV-CyP/DSPE-PEG_2000_	625	1.18 × 10^9^	—	461.3 (s.c./30 µg)	1	Screening of antitumor drugs inducing ICD	63
AIE/B-AGL-HCPT NPs	B-AGL-HCPT (AGL-2)/(TPE DPA)_2_-Py/DSPE-PEG_2000_	670	—	118.5	—	—	Drug release and tracking in immunotherapy	64
AGL AIE NPs	AGL-5/TPE-Ph-DCM/Lipid-PEG_2000_	620	1.0 × 10^7^	48	—	0.7	Image-guided cancer surgery	44
TPT-DCM/AGL NPs	AGL-3/TPT-DCM/DSPE-PEG_2000_	630	—	—	—	1.6	Image-guided cancer surgery	61

**1,4-Oxathiin and 1,4-dioxin**								
ALNPs	DO/NCBS/PFVA/PEG-*b*-PPG-*b*-PEG	780	2.4 × 10^9^	3	2922 (i.v./95.3 µg)	—	Tumor imaging	45
UCANPs@RAW	SO/Eu(TTA)_3_Phen/PdPc(OBu)_8_/polystyrene	620	—	3.3	258 (i.v./2 × 106 cells)	—	Visualization of acute brain inflammation	47
AGNP	CUEM/SiPc/carboxylated polystyrene	445	—	0.04	131 (s.c./150 µg)	—	Lateral flow immunoassay	46
PA-gel	SO/PdPc(Obu)_8_/Eu(TPPO)_2_(b-NTA)_3_	613	—	0.04	—	—	Arterial embolism real-time imaging	48
TPP-DO NPs	TPP-DO/DSPE-PEG_2000_	655	4.0 × 10^8^	2	3600 (i.v./40 µg)	6	Tumor imaging	49

**Rubrene**								
MANS	Rubrene/Ir-OTf/F-127	560	6.8 × 10^5^	2	4 (i.p./210 mg kg^−1^)	—	Imaging cisplatin-induced acute kidney injury through detection of O_2_˙^−^	51

**Porphyrin**								
Ppa-FFGYSA	Ppa	760	3.2 × 10^5^	˃ 60	215.1 (i.v./500 µM)	1	*In situ* breast tumor imaging	52
Ch-NPs	Ce4/PEG-*b*-PPG-*b*-PEG	660	1.4 × 10^5^	90	690 (s.c./40 µg)	1	Microscopic peritoneal metastatic tumor imaging	53

**Hemicyanine derivative**								
MAP-O_2_˙^−^ and MAP-LAP	HD-Br	721	—	1	—	—	APAP-induced early drug-induced hepatotoxicity imaging	54
RAN	MAS-pH/PMSA/DSPE-PEG	690–770	—	1.6	—	—	Monitoring tumor glycolysis levels	55

**Methylene blue derivative**								
SAN-MO	mMB/PEG-*b*-PPG-*b*-PEG/DSPE-PEG_2000_	680	2.1 × 10^7^	4	160 (s.c./10 µg)	5	Real-time monitoring of LPS-induced inflammatory processes	56

**Boron dipyrromethene derivative**								
BDIS-NPs	BDIS/PEG-*b*-PPG-*b*-PEG	780	2.0 × 10^6^	1.5	80.1 (i.v./10 µg)	4	Ultra-early monitoring of acute lung injury and H2S level visualization of schizophrenia	58

**Ultrasound-activated afterglow probes**								
SNAPs, SNAP-M	Pro-DPAs/NCBS/PEG-*b*-PPG-*b*-PEG	780	5.9 × 10^6^	1.83	72.2 (i.v./50 µg)	4	M1 macrophage imaging	30
Q-SNAP	DAPs/NCBS/PEG-*b*-PPG-*b*-PEG DSPE-PEG1000	720	2.0 × 10^6^	5	58.3 (i.v./2 mg kg^−1^)	4	T Cell imaging	60
TD-NPs	TD/DSPE-PEG	640	—	3	—	2.2	Tumor imaging	31

**Radio-activated afterglow probes**								
MRAP	IDPAs/cRGD/Cit-Val	770	2.1 × 10^6^	18.4	—	15	Deep-tissue cancer theranostics	32
tRANP	CzTPN/NIR775/cDTDP/DSPE-PEG2000	780	9.0 × 10^5^	4.8	207 (i.v./25 µg)	15	Guided radiodynamic therapy	33

a *λ*_em_ is the maximum afterglow emission wavelength. *t*_1/2_ is the half-life. SBR is the signal-to background ratio, and administration/dose. PD is the penetration depth.

Currently, afterglow imaging probes based on the above substrates have been applied in various biomedical fields: (1) disease visualization, such as tumor and lymph node imaging,^35–37,39^ drug-induced liver injury monitoring,^35,54^ acute inflammation visualization,^47,56^ and dynamic tracking of acute kidney injury;^51^ (2) therapeutic navigation, such as image-guided cancer surgery,^44,52,53,61^ transcatheter arterial embolization (TAE) assessment,^48^ and real-time monitoring of photothermal/photodynamic therapy;^38,42,43^ (3) pharmaceutical research, involving drug delivery, tracking, and screening.^62–64^ For *in vitro* diagnostics, such probes have been successfully used for the ultrasensitive detection of cancer exosomes^65^ and early pregnancy diagnosis.^46^ Through molecular engineered modifications, afterglow probes have been developed that specifically recognize biomarkers, such as cysteine, hydrogen sulfide (H_2_S),^66,67^ peroxynitrite (ONOO^−^)^30,63,64^ and hydrogen peroxide (H_2_O_2_),^33,62^ granzyme B (GZMB).^31,60^ Collectively, this research provides new tools for accurate disease diagnosis. These diversified applications underscore the potential of afterglow imaging probes for disease diagnosis (Fig. 1).

With this perspective, we systematically summarize research progress toward organic afterglow imaging probes, concentrating on molecular design, luminescence mechanism and biomedical applications: first, we classify the afterglow probes according to the excitation source (photo-activated probes, ultrasound-activated probes and X-ray-activated probes) and molecular substrates (*e.g.* PPV, thiophene-based polymers, AEEs, and porphyrins) to provide a design basis for these probes (Fig. 2). Second, we summarize the core principles of the molecular design: optimizing the intramolecular charge transfer effect using donor–acceptor structures that serve to extend the afterglow wavelength through increasing the conjugation of the system or introducing rigid aromatic rings to reduce non-radiative processes. We also discuss nano-engineering to enhance the stability of the probes and enrichment in tumour regions. Third, we discuss the cascade mechanisms of afterglow luminescence: where external energy (light/ultrasound/X-ray) excites the probe to generate ^1^O_2_, which oxidizes the molecular skeleton (*e.g.*, C=C bond) to form a dioxetane intermediate, which spontaneously decomposes to release chemical energy and afterglow luminescence. We also explore the role of energy transfer pathways (*e.g.*, FRET, CRET) to modulate the intensity and wavelength of the afterglow emission. Finally, we discuss the application of afterglow imaging probes in biomedicine and evaluate these innovative molecular tools for precision medicine (Fig. 2). We believe that afterglow luminescent probes can provide for improved medical treatments by overcoming the challenges of penetration depth and biosafety, and that these attributes will ultimately facilitate the successful transition of afterglow probes from the laboratory to the clinic.

## 2. Photo-activated afterglow probes: molecular design and biomedical applications

### 2.1. Polyphenylene vinylene-based afterglow probes: from nano-engineering to therapeutic integration

**Fig. 3:**
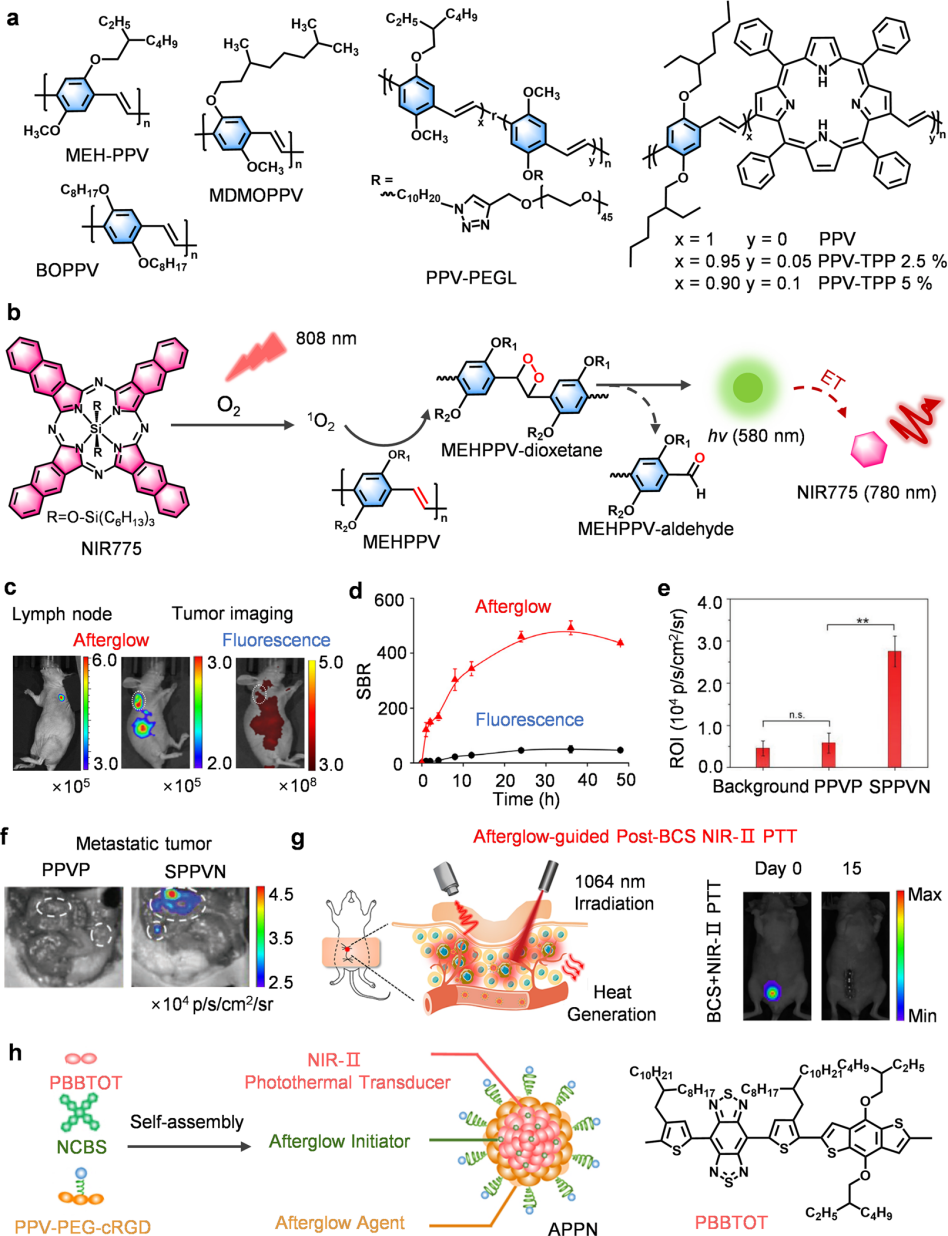
PPV-based afterglow probes for biomedical diagnosis. Schematic diagram showing: (a) chemical structures of PPV-based afterglow substrates and (b) afterglow luminescence mechanism. (c) Representative afterglow images of lymph node sites in mice, and (d) SBR. (e) Afterglow intensity of region of interest (ROI), and (f) afterglow images of peritoneal metastatic tumors in 4T1 tumor-bearing mice after injection of SPPVN or PPVP. (g) Afterglow-guided post-BCS NIR-Ⅱ photothermal therapy (PTT), and representative afterglow images of APPN. Panels b–d reprinted with permission from ref. 35. Copyright 2017 Springer Nature. Panels e and f reprinted with permission from ref. 36. Copyright 2018 Wiley. Panels g and h reprinted with permission from ref. 38. Copyright 2023 American Chemical Society.

The structural flexibility and optoelectronic properties of polyphenylene vinylene (PPV) derivatives establish a solid foundation for next-generation afterglow probes.^68–70^ As pioneered by Rao *et al.*, semiconducting polymer nanoparticles (SPNs) derived from MEH-PPV uses the styrene-linked polymer backbone to achieve unprecedented long afterglow durations exceeding 1 hour post-excitation, a performance metric unattainable with conventional fluorescent materials.^34^ This arises from the design of alkoxy-functionalized PPVs (such as BOPPV and MDMOPPV), whose increased oxidative sensitivity at vinyl linkages facilitates the formation of singlet oxygen–mediated dioxetane intermediates (Fig. 3a).^35^

The evolution of the field underscores a shift from passive encapsulation to active covalent engineering. Compared with conventional single-component afterglow probes, which often suffer from limited tissue penetration and suboptimal brightness, Pu *et al.* demonstrated that co-assembling MEH-PPV with photosensitizers (*e.g.*, NCBS) *via* nanoprecipitation enables dual-level optimization (Fig. 3b).^35^ Mechanistically, energy from MEH-PPV is transferred to NCBS *via* FRET, achieving a red-shifted emission (780 nm), while NCBS itself enhances ^1^O_2_ generation, boosting afterglow brightness. By further fine-tuning the NCBS doping ratio, they achieved *in vivo* imaging of mouse tumors and lymph nodes (Fig. 3c and d). Objectively, this work illustrates that rational photosensitizer integration not only improves signal intensity and spectral properties but also expands the functional applicability of afterglow probes for deep-tissue imaging, highlighting potential advantages over conventional single-component systems.

Further breakthroughs emerged using covalent modifications. Compared with traditional amphiphilic PPV preparations, which often suffer from large particle size, low surface functional density, and limited afterglow brightness, covalent modification of PPV backbones with hydrophilic PEG enables the formation of self-assembled PPV nanoparticles (SPPVNs) with reduced hydrodynamic diameter (∼24 nm), enhanced PEG surface density, and a 6-fold increase in tumor afterglow intensity (Fig. 3a).^36^ Following intravenous injection into 4T1 tumor-bearing mice, SPPVNs achieved ∼6-fold higher afterglow in tumors than unmodified PPVP, and allowed for the detection of tumors as small as 1 mm^3^ (Fig. 3e and f). From a strategic perspective, this covalent architectural control balances biodegradability with tunable ^1^O_2_ kinetics and controlled decomposition of chemiluminescent intermediates, marking a transformative strategy in PPV-based probe design. Building on these structural and photophysical advances, PPV-based probes have been extended to multifunctional platforms. For example, Zhen *et al.* developed an afterglow/photothermal bifunctional probe (APPN) (Fig. 3h),^38^ which not only accurately locates minimal residual lesions *via* high-SBR (∼150) afterglow imaging but also guides precise photothermal ablation, achieving complete tumor elimination. Consequently, afterglow signals in the tumor region significantly diminished, and recurrence rates were substantially reduced 15 days post-surgery (Fig. 3g). These studies illustrate that covalent engineering of PPV backbones offers advantages in brightness, targeting precision, and therapeutic integration over conventional approaches, providing a robust platform for imaging-guided cancer therapy.

**Fig. 4:**
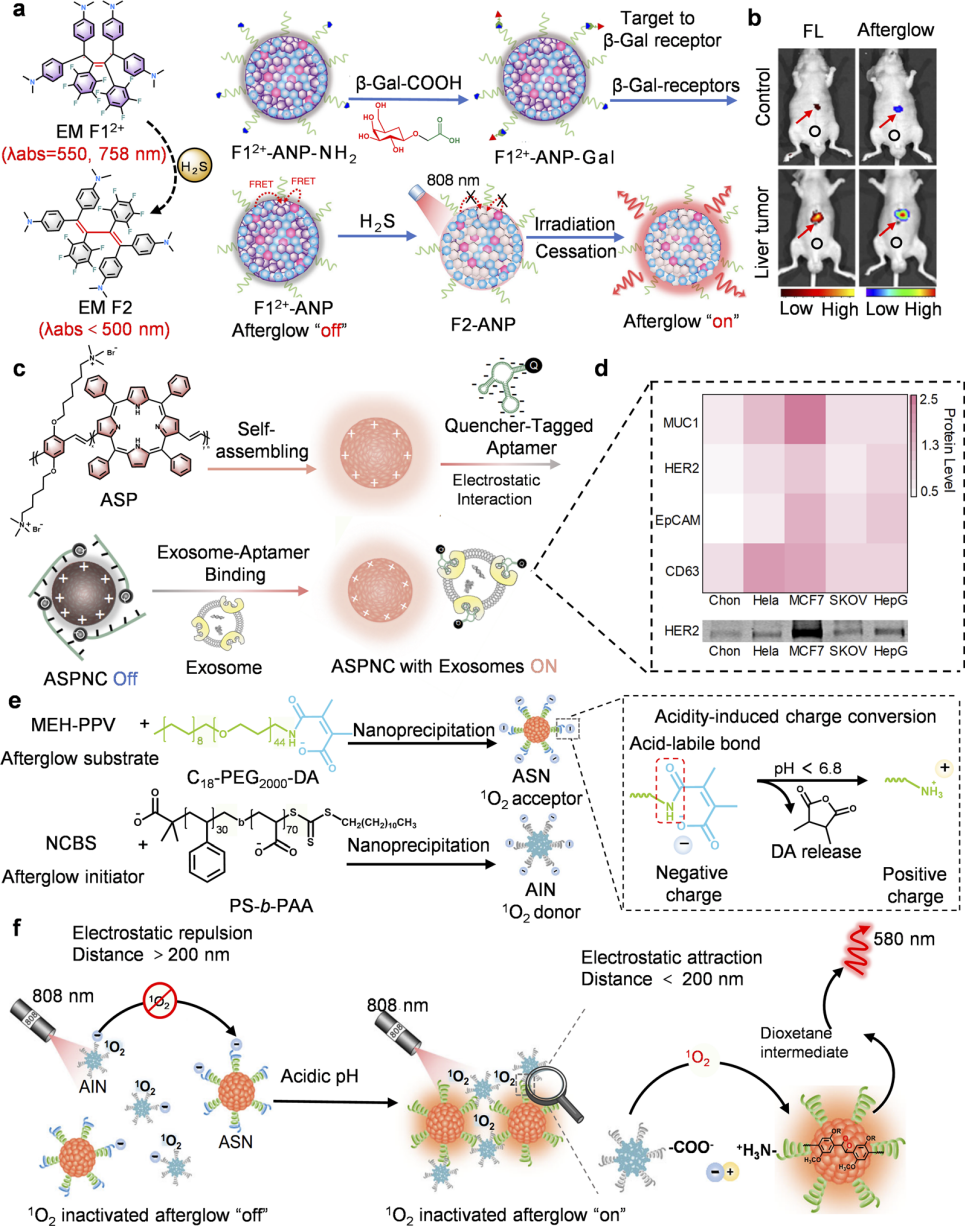
PPV-based activatable afterglow probes for bioimaging and *in vitro* diagnosis. (a) Schematic diagram showing construction and the afterglow activation mechanism of F1^2+^-ANP-Gal. (b) Representative afterglow and fluorescence images of an orthotopic HepG2 tumor after intravenous injection of F1^2+^-ANP-Gal. (c) Schematic diagram illustrating the construction and afterglow activation mechanism of ASPNC. (d) Heat map of afterglow recovery ratio per unit concentration, showing the expression levels. (e) Schematic diagram of construction and (f) afterglow activation mechanism of ALCNs. Panels a and b reprinted with permission from ref. 66. Copyright 2020 Springer Nature. Panels c and d reprinted with permission from ref. 65 Copyright 2019 Wiley. Panels e and f reprinted with permission from ref. 39. Copyright 2024 Springer Nature.

Despite notable achievements, current MEH-PPV afterglow probes face two key limitations: first, reliance on passive PEG targeting results in low tumor accumulation efficiency; second, the “always-on” imaging mode induces background interference, which limits the effective delivery and imaging sensitivity. Even if “off-on” probes can suppress background noise, existing technologies lack precise activation strategies suitable for *in vivo* environments. Notably, activatable afterglow probes can release signals by deactivating quenching in the presence of specific biomarkers, significantly enhancing detection sensitivity and targeting. For instance, Ye's team developed the H_2_S-detecting probe F1^2+^-ANP through structural optimization and dynamic electron transfer strategies.^66^ Its mechanism relies on the electrochromic molecule F1^2+^ suppressing afterglow signals in its initial state (off state) (Fig. 4a).^71^ Then upon H_2_S reduction of F1^2+^, this suppression is removed, specifically activating NIR afterglow (the on state), ultimately achieving high SBR (∼124.5) imaging of *in situ* liver tumors (Fig. 4b).

Traditional nanoprecipitation suffers from uneven spatial distribution, which facilitates photosensitizer leakage and thus undermines stability, while also lowering FRET efficiency. To overcome these limitations, Pu's team developed a covalent doping strategy that involved linking TPP directly to the PPV backbone, resulting in the formation of covalently embedded PPV-TPP polymers (Fig. 3a).^37^ This approach effectively improves the uniform distribution of photosensitizers within the polymer chains, avoids phase separation caused by physical mixing, and prevents TPP leakage through covalent fixation. The net result is enhanced nanoparticle stability. Furthermore, Pu's team designed an afterglow imaging nanocomplex (ASPNC) by conjugating an afterglow polymer (ASPN) with a quencher (BHQ-2)-labeled nucleic acid aptamer (Apt_CD63_) (Fig. 4c).^65^ Replacing the aptamer sequence gave rise to an ASPNC probe permitting the orthogonal detection of multiple exosome markers (Fig. 4d).

Despite afterglow probes achieving breakthroughs in biomarker response and signaling regulation, the complexity of the tumor microenvironment requires higher precision. Although the above dynamic electron transfer strategies and covalent doping techniques have significantly enhanced the sensitivity and specificity, precise signal activation in the acidic tumor microenvironment remains a key challenge. Accordingly, Miao *et al.* developed pH-activated up-conversion afterglow probes (ALCNs) (Fig. 4e).^39^ The core innovation lies in the charge reversal-mediated dynamic nanoparticle assembly mechanism: it comprises negatively charged afterglow initiators (AIN) and acid-sensitive group (2,3-dimethylmaleic anhydride, DA)-modified afterglow substrates (ASN). As a result, at physiological pH, DA becomes negatively charged on the ASN, causing electrostatic repulsion with the similarly negatively charged AIN. This prevents them from approaching each other, thereby blocking the effective energy transfer of ^1^O_2_ from AIN to ASN (Fig. 4f). In the acidic tumor microenvironment, the surface charge of ASN reverses and aggregates with AIN *via* electrostatic attraction, activating the afterglow signal at the tumor site.

The above advances notwithstanding, persistent barriers in background interference and tumor-specific targeting demand deeper exploration of electronic effects and responsive mechanisms. Future directions could involve: (1) spectral optimization: extending emission into the NIR-IIb window (1500–1700 nm) in order to minimize tissue scattering and autofluorescence. Moreover, covalent doping of photosensitizers (*e.g.*, TPP) into PPV chains, as demonstrated in SPN2.5, can offer significant afterglow amplification and NIR emission *via* enhanced FRET and ^1^O_2_ production. Reaching beyond 1000 nm will require novel chromophore designs exploiting excited-state intramolecular proton transfer (ESIPT) or aggregation-induced emission (AIE). (2) Dynamic biofunctionalization: regulating hydrodynamic diameters (5–20 nm) should be coupled with surface engineering. Dynamic PEGylation or stimuli-responsive ligands can be employed to extend plasma half-life and suppress off-target signals, thereby enhancing target-to-background ratios in complex microenvironments. (3) Probes with precisely activable architectures: future innovations should transcend passive encapsulation, thus multifunctional crosslinkers or supramolecular assemblies (*e.g.*, pH-responsive ALCNs) that leverage tumor-specific triggers (*e.g.*, charge reversal) for precise activation will be essential.

### 2.2. Thiophene-based afterglow probes: afterglow signal amplification and photodynamic therapy

**Fig. 5:**
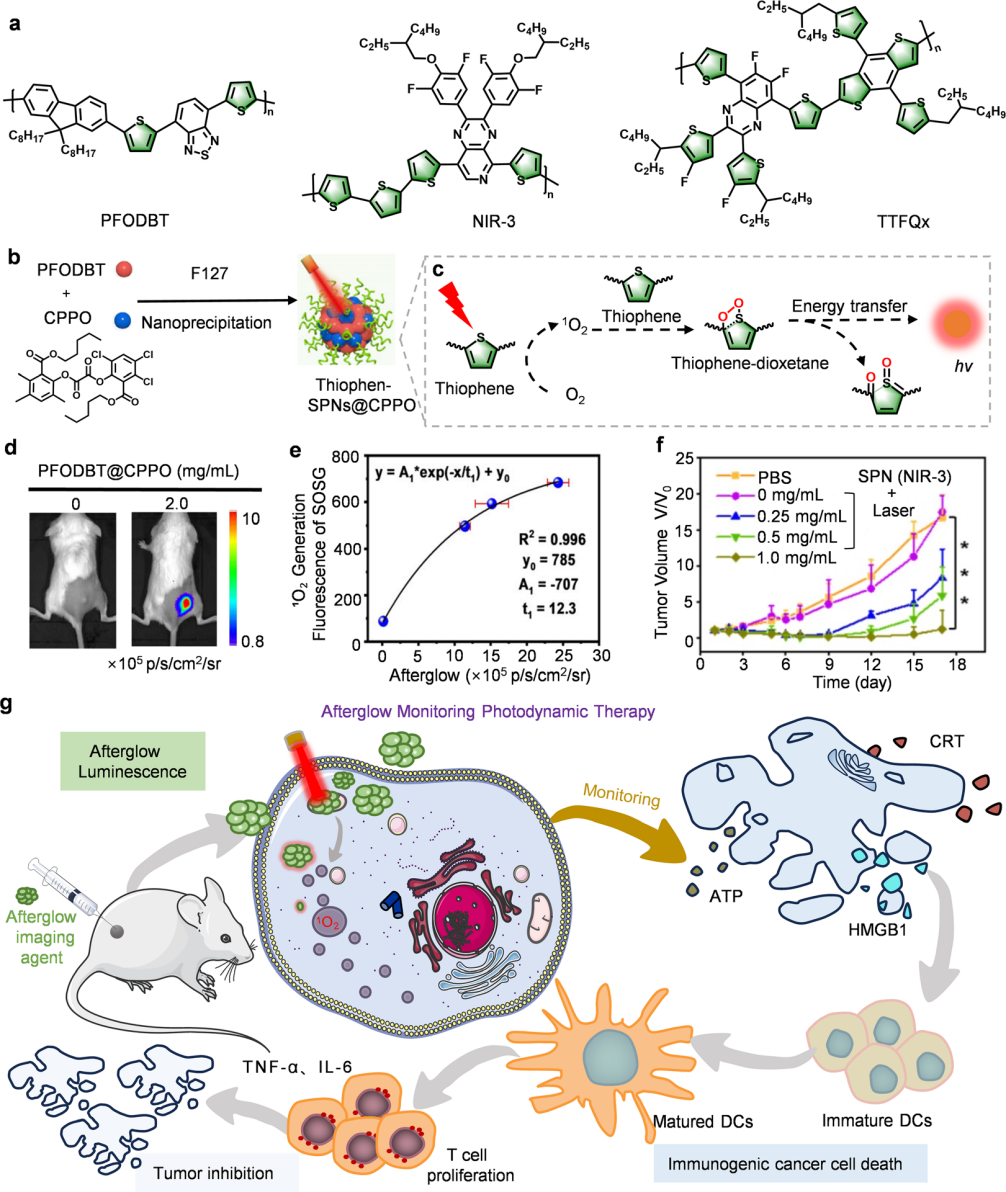
Thiophene-based afterglow probes for afterglow imaging-guided PDT. Schematic diagram showing (a) chemical structures of thiophene-based afterglow molecular substrate. (b) Structural composition, and (c) afterglow luminescence mechanism of Thiophen-SPNs@CPPO. (d) Representative afterglow images of mice bearing 4T1 tumors after injection of Thiophen-SPNs@CPPO. (e) Correlation between afterglow intensity of Thiophen-SPNs@CPPO and ^1^O_2_ yield. (f) Tumor growth curves of CT26 tumor-bearing mice after injection of NIR-3. (g) Schematic diagram of afterglow-mediated therapeutic monitoring. Panels b–e reprinted with permission from ref. 42. Copyright 2021 Wiley. Panel f reprinted with permission from ref. 43. Copyright 2022 Ivyspring International Publisher.

Thiophene-based semiconducting polymers represent a crucial class of functional materials, whose unique electronic architecture featuring electron-rich sulfur heteroatoms and planar conjugated backbones confers exceptional advantages in afterglow imaging.^72–74^ Importantly, the sulfur atom intrinsically narrows the singlet-triplet energy gap (ΔEST), which accelerates intersystem crossing (ISC) to populate triplet states and generate cytotoxic ^1^O_2_. Moreover, the unsaturated C=C bonds adjacent to sulfur creates oxidation-vulnerable sites central to afterglow mechanisms. As illustrated in Fig. 5c, ^1^O_2_-mediated formation of transient thiophene-dioxetane intermediates generates afterglow luminescence through exergonic decomposition, while extended π-conjugation amplifies signals *via* enhanced intermolecular π–π stacking. This structural framework enables spectral tuning by modifying the polymer topology, appending substituents, or modulating conjugation length, which allows red-shifted absorption/emission for the imaging of deep-tissue pathologies.

Generally intrinsic afterglow efficiency remains constrained. To overcome this limitation Zhang *et al.* pioneered a transformative signal amplification strategy by integrating the chemiluminescent substrate (CPPO) into a matrix consisting of the semiconducting polymer (PFODBT) giving rise to PFODBT@CPPO (Fig. 5a and b).^42^ This bifunctional system was found to support a self-propagating cycle: Photogenerated ^1^O_2_ oxidizes both of the thiophene units and CPPO. Subsequent, energy transfer from the decomposing intermediates excites PFODBT, generating afterglow and regenerating the ^1^O_2_, forming a feedback loop that boosts luminescence and suppresses background interference (Fig. 5d and e). Beyond imaging, this system provides potent antitumor immunity: excess ^1^O_2_ induces immunogenic cell death, prompting the induction of damage associated molecular patterns (DAMPs) that reverse immunosuppression (Fig. 5g). Crucially, the probe upregulates pro-inflammatory cytokines (TNF-α, IL-6), thus enabling a dual-theranostic milestone, *i.e.* the monitoring of ROS dynamics and immune reprogramming during therapy.

Despite these advances, fundamental limitations in afterglow brightness and photodynamic efficacy persist. Addressing this, Song *et al.* engineered a semiconducting polymer probe (NIR-3) by introducing a strong electron acceptor pyrido pyrazine and optimizing thiophene donor geometry (Fig. 5a).^43^ This design maximizes intramolecular charge transfer (ICT), and reduces ΔEST to enhance the ISC efficiency while red-shifting the afterglow to beyond 800 nm. By introducing more thiophene structures into the main chain to provide sufficient reaction sites for afterglow generation, the afterglow intensity of SPN (NIR-3) was optimized to the point where it was superior to that of previous-generations of organic afterglow probes, such as MEHPPV and PFODBT. The efficient ^1^O_2_ generation and NIR emission reduce tissue scattering and background interference, thus enabling deep phototherapeutic penetration. This advantage is evidenced by dose-responsive tumor inhibition (0.25 → 1.0 mg mL^−1^, Fig. 5f) and high-contrast afterglow imaging under 2 cm-thick of chicken tissue.

To advance clinical translation of thiophene-based afterglow platforms from the current state of molecular optimization, we envision the following key steps: (1) ΔEST reduction and signal multiplication: tailoring energy-levels *via* heavy-atom effects or triplet–triplet annihilation systems may further amplify afterglow quantum yields >10-fold. Likewise, hybrid energy-relay architectures, *e.g.*, triplet sensitizer-acceptor dyads, can spatially separate the absorption and emission processes, thereby minimizing self-absorption and enhancing the overall afterglow efficiency. (2) Microenvironment-responsive afterglow: Integrating stimuli-cleavable linkers *e.g.*, pH-/enzyme-labile bonds, into the backbones of afterglow probes will enable exclusive activation in the tumor microenvironment, thus overcoming off-target signals. Furthermore, synergistic multi-stimuli gating (*e.g.*, H_2_O_2_ + hypoxia) can further enhance the specificity. (3) Complete theranostics: real-time afterglow feedback must guide adaptive therapeutic modulation, particularly at the tumor margins. Thus, if afterglow sensors can be embedded within implantable bioelectronic interfaces, synchronous imaging and iontophoretic drug modulation can be expected. (4) Acousto-optical fusion: merging ultrasound techniques with afterglow probes will transcend photon penetration limits. Clinically diagnostic ultrasound systems could trigger/read nanosecond afterglow pulses, whereby mapping the microlesions currently invisible to magnetic resonance imaging (MRI). (5) Hybrid clinical translation: conjugating afterglow moieties to FDA-approved agents *e.g.*, indocyanine green (ICG) or porphyrins, may offer rapid approval through regulatory pathways.

### 2.3. Adamantylidene enol ether-based afterglow probes: tumor microenvironment response and drug release visualization

**Fig. 6:**
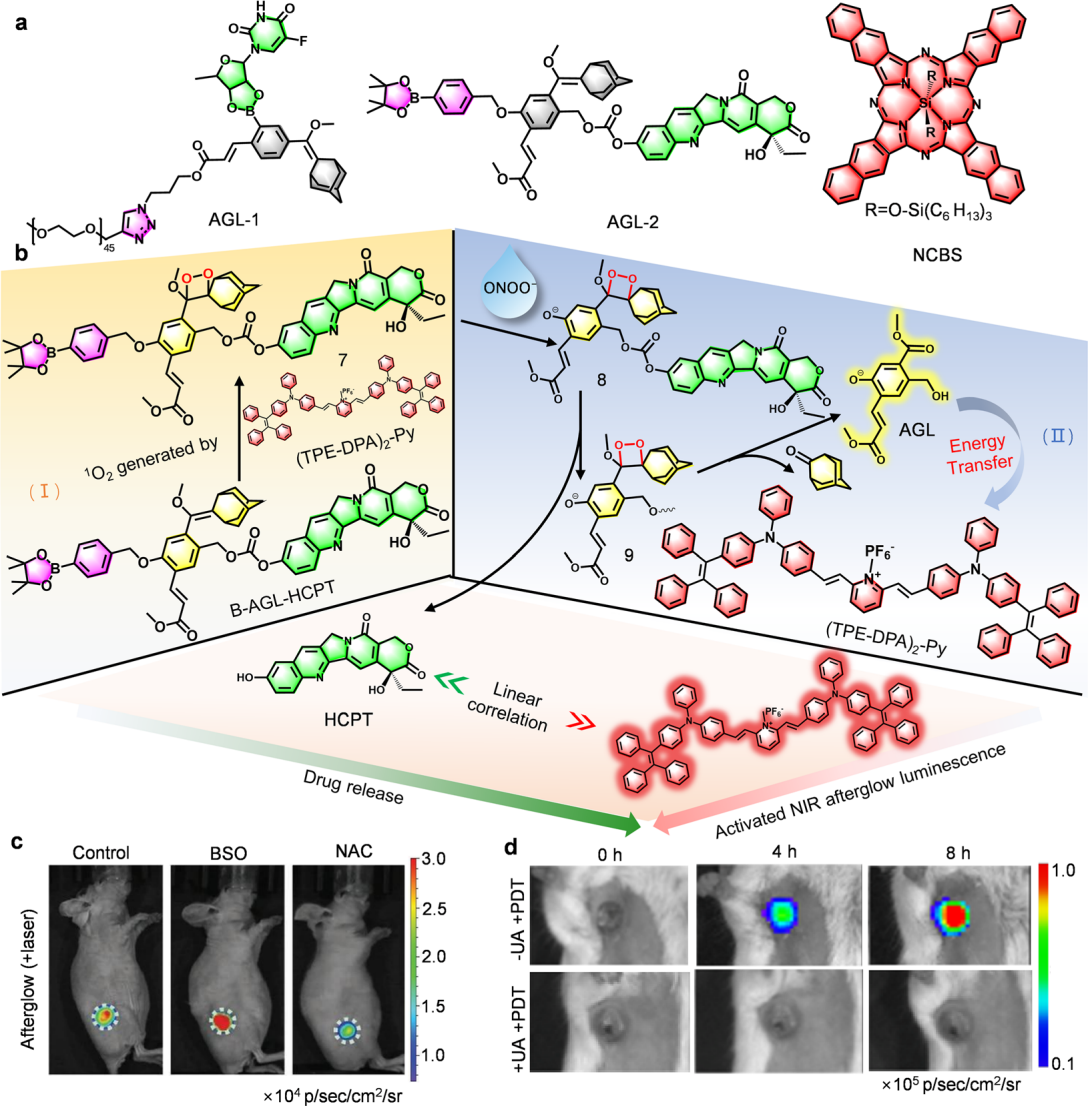
AEEs-based afterglow probes for drug delivery and tracking. Schematic diagram showing (a) Chemical structures of the AEEs-based afterglow molecular substrates, and (b) afterglow activation and drug release. (c) Representative afterglow images of 4T1 breast cancer tumors in 4T1 tumor-bearing mice after injection of APtN. BSO: glutathione biosynthesis inhibitor; NAC: ROS scavenger. (d) Representative afterglow images after injection of AIE/B-AGL-HCPT NP. Panel c reprinted with permission from ref. 62. Copyright 2019 Wiley. Panels b and d reprinted with permission from ref. 64. Copyright 2022 Wiley.

**Fig. 7:**
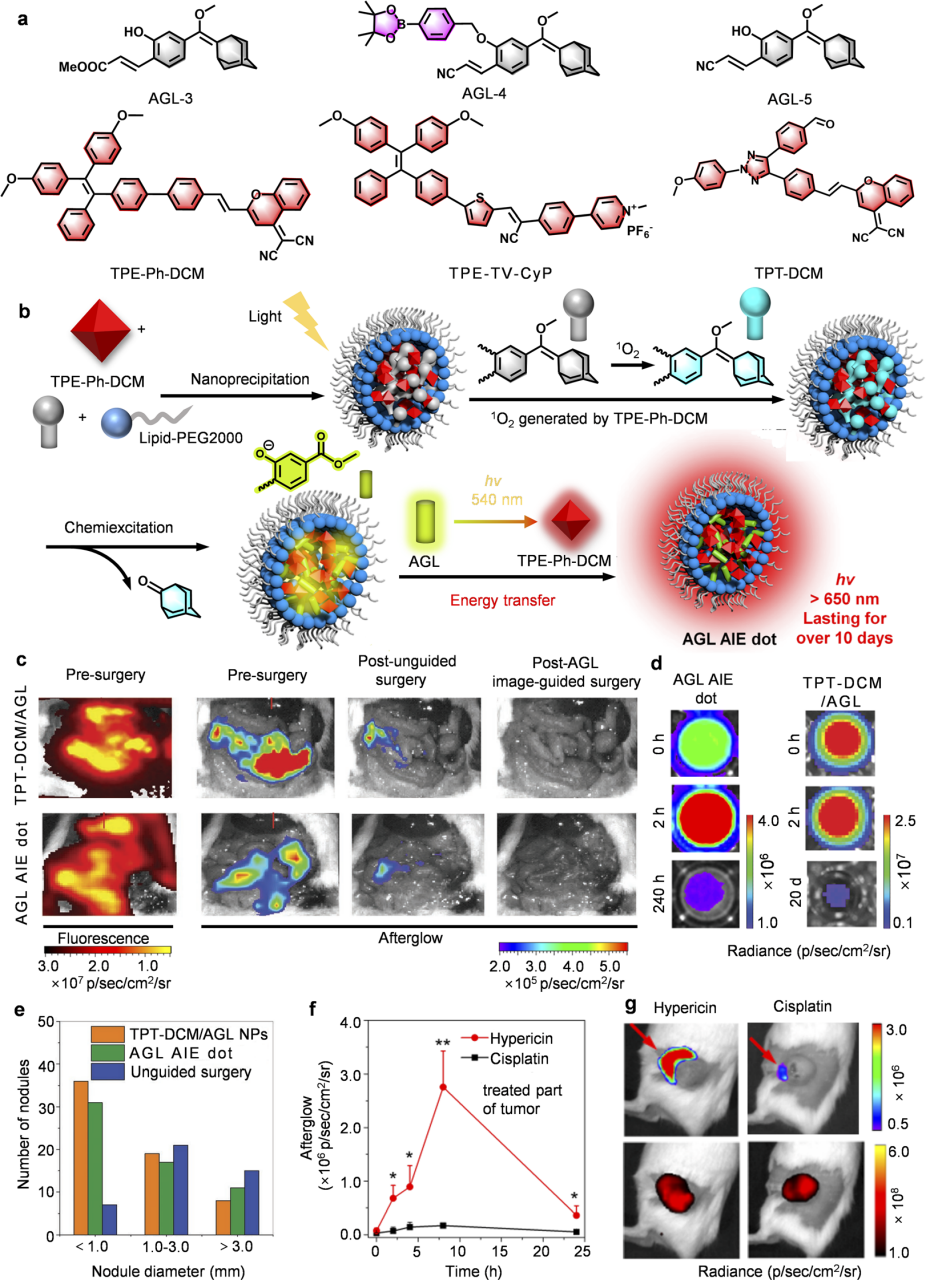
AEEs-based afterglow probes for drug screening and surgical navigation. (a) Chemical structures of AEEs-based afterglow molecular substrates. (b) Schematic diagrams showing afterglow luminescence. (c) Representative fluorescence and afterglow images before and after tumor resection following injection of AGL AIE dots or TPT-DCM/AGL in peritoneal carcinomatosis-bearing mice. (d) Schematic diagram of afterglow luminescence, and (e) number of tumors in Balb/c mice bearing peritoneal 4T1 tumors. (f) The afterglow intensity, and (g) representative afterglow images of 4T1 tumor-bearing mice post injection of PA-AGL NPs in hypericin or cisplatin-treated mice. Panel b reprinted with permission from ref. 44. Copyright 2019 American Chemical Society. Panels c–e reprinted with permission from ref. 61. Copyright 2023 Wiley. Panels f and g reprinted with permission from ref. 63. Copyright 2022 American Chemical Society.

Adamantylidene enol ethers (AEEs, *i.e.*, the enol ether precursors of Schaap's dioxetanes) act as flexible key building blocks in dioxetane chemistry, and are widely used in the design of high-performance afterglow luminescent probes.^75–79^ Molecular engineering synergizes CIEEL with energy transfer (*e.g.*, CRET) to achieve controlled red-shifting of the afterglow emissions through precise energy-level modulation. While AEEs depend on exogenous ^1^O_2_ for dioxetane formation, this requirement enables unique advantages: modular stimulus-responsive design *via* flexible coupling with ^1^O_2_ sources and superior microenvironment sensing by dynamic capture of ROS activity with spatiotemporal precision, distinguishing AEEs from autonomous chemiluminophores for targeted biosensing applications. Recent advancements reflect the following:

*Theranostic integration that goes beyond conventional drug delivery to allow for both imaging and therapy*. Compared with conventional drug delivery systems that rely on passive reporting, Pu's team have pioneered a covalent fusion of AEE-based afterglow substrates (AGL-1) with prodrugs (5-DFUR) *via* H_2_O_2_-cleavable linkers, creating a bioresponsive theranostic platform (Fig. 6a).^62^ By establishing a quantitative correlation between afterglow intensity and drug release, their APtN assembly transforms optical signals into real-time pharmacokinetic readouts (Fig. 6c), offering a significant advance over traditional methods that cannot monitor drug dynamics *in situ*. While groundbreaking, this system also points to the next frontier: adaptive therapy, where afterglow signals could autonomously guide treatment parameters such as light-dosing intervals. In a parallel strategy, Ding's ONOO^−^-activated probe (AIE/B-AGL-HCPT NP) combines an ONOO^−^-responsive AGL-2 prodrug with a NIR photosensitizer ((TPE-DPA)_2_-Py), establishing a self-amplifying circuit, *i.e.* “ICD → ONOO^−^ → drug release → ICD enhancement” (Fig. 6b and d),^64^ which exemplifies the versatility of AEEs for multi-step therapeutic feedback. Furthermore, Ding's ONOO^−^/pH dual-responsive probe (PA-AGL NP) extends afterglow luminescence up to 14 days, simultaneously capturing neutrophil infiltration and microenvironment acidification, thereby enabling rapid evaluation of ICD drug efficacy (Fig. 7f and g).^63^ As such, these platforms collectively demonstrate that covalent integration, dual/multi-responsive design, and signal amplification can convert afterglow imaging from passive reporting into active, real-time pharmacological feedback. They highlight a clear advantage over single-analyte approaches, providing a robust framework to decipher complex microenvironmental cues (ROS, pH, ATP) and guide the design of the next-generation of adaptive theranostic systems.

*Balancing penetration and sensitivity are essential for advancing surgical navigation*. Compared with conventional fluorescence imaging limited by rapid signal decay and low contrast, Ding's AGL AIE dots achieved exceptional persistence (>10 days) and a tumor-to-liver contrast ratio of 34.2-fold in peritoneal carcinomatosis-bearing mice, enabling precise submillimeter tumor resection not possible using fluorescence guidance (Fig. 7b).^44^ Building on this foundation, Liu *et al.* replaced the traditional TPE with a triazole-based TPT core, achieving an optimal balance between molecular distortion and conjugation. The resulting TPT-DCM/AGL nanoparticles exhibited a higher molar extinction coefficient and improved ^1^O_2_ generation, while synergistic CRET with Schaap's dioxetane precursor (AGL-5) and AIE effects enabled deep tissue penetration (1.6 cm) and an exceptional SBR of 187 : 1 (Fig. 7c and d).^61^ Critically, this work exemplifies how the convergence of molecular engineering and energy-transfer amplification can redefine the scope of afterglow imaging from an exploratory visualization technique to a clinically consequential modality. The demonstrated ability to guide the complete removal of 37 submillimeter tumor nodules (*versus* 30 with AGL AIE dots) with no residual lesions (Fig. 7e) provides not only empirical validation but also a benchmark for translational efficacy. More importantly, it substantiates the practical feasibility of afterglow-guided surgery, underscoring how rational probe design can translate photophysical innovation into measurable therapeutic gain. These results underscore how rational photophysical design can bridge molecular innovation and clinical performance, setting a new benchmark for precision oncologic imaging and guided surgery. Despite these advances, significant challenges remain, particularly in the design of emitters that can simultaneously maximize extinction coefficients, ROS quantum yields, and CRET efficiency while preserving biocompatibility.

Based on mechanistic insights, clinical needs, and recent advances in AEE chemistry, we foresee three future priorities for intelligent cell-scale resection. First, subcellular resolution: current submillimeter detection must evolve toward single-cell and microvessel mapping. The integration of super-resolution afterglow microscopy with tumor-targeted AEE derivatives could potentially achieve ∼50 µm intraoperative precision, and this would enable nerve-sparing oncologic surgery. Second, dynamic tissue compensation: vascular pulsations and respiratory movements introduce millisecond-scale artifacts in imaging, making hemodynamic-adaptive detection essential, that can be achieved using gated acquisition or motion-triggered signaling mechanisms, which will achieve motion artifact suppression and enable real-time boundary tracking. Third, on-demand clearance: existing probes carry risks of inflammatory sequelae. This underscores the need for activatable probes with controlled clearance profiles.

### 2.4. 1,4-Dioxin and 1,4-oxathiin-based afterglow probes: molecular fusion strategies and *in vitro* diagnostics

**Fig. 8:**
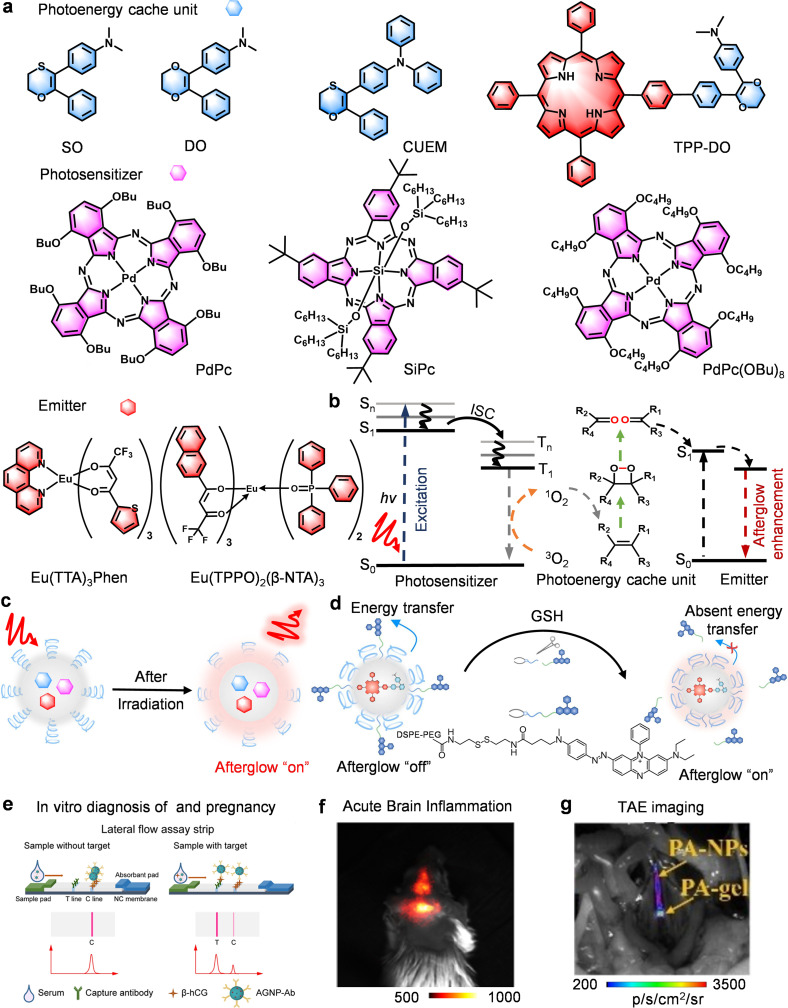
DO/SO-based afterglow probes for biomedical applications. (a) Chemical structures of DO/SO-based afterglow molecular substrates, and (b) afterglow luminescence mechanism and energy transfer mechanism, and (c) afterglow imaging illustration. (d) Schematic diagrams showing the structure of GSH-activatable afterglow probes and afterglow turn-on. (e) Schematic diagrams illustrating the detection principle of LFIA strips based on AGNP-Ab and interpretation. (f) Representative afterglow image of acute encephalitis after injection of UCANPs@RAW. (g) Representative afterglow image of arterial embolism after injection of PA-NPs. Panels b–d reprinted with permission from ref. 49. Copyright 2023 Wiley. Panel e reprinted with permission from ref. 46. Copyright 2022 Wiley. Panel f reprinted with permission from ref. 47. Copyright 2022 American Chemical Society. Panel g reprinted with permission from ref. 48. Copyright 2021 Wiley.

The development of afterglow luminescence nanoparticles (ALNPs) based on 1,4-dioxin (DO) and 1,4-oxathiin (SO) scaffolds represents a significant step forward in optical bioimaging (Fig. 8a).^80^ In contrast to conventional fluorescent agents that require continuous excitation, these scaffolds exploit vinyl bonds as intrinsic energy reservoirs, enabling sustained photon emission after initial excitation. Early work by Pu and colleagues established a tripartite design comprising afterglow initiator (photosensitizer, PS), afterglow substrate (energy cache unit, ECU), and afterglow relay unit (emitter), which together harness ^1^O_2_-mediated dioxetane formation to generate delayed luminescence (Fig. 8b and c).^45^ Following this, many afterglow imaging probes based on DO and SO have been reported.^47,48^ Building on this foundation, recent advances have focused on molecular-level strategies to overcome energy-transfer bottlenecks and amplify afterglow efficiency.

*Molecular fusion: advancing energy confinement*. This strategy emerged from intramolecular engineering approaches designed to overcome energy-transfer bottlenecks. Compared with conventional physically blended systems prone to energy loss and limited ^1^O_2_ generation, Li *et al.* developed a covalently fused CUEM molecule integrating a triphenylamine emitter with an energy-caching unit, achieving a 162-fold afterglow enhancement and a record quantum yield of 2.59%.^46^ This molecular fusion suppresses non-radiative decay and boosts ^1^O_2_ efficiency, outperforming traditional blends. Extending this concept, Miao *et al.* created an “all-in-one” NIR afterglow probe (TPP-DO) by covalently linking TPP (PS and emitter) with DO (ECU), increasing afterglow 56-fold and enabling ultrasensitive tumor imaging achieving high-SBR imaging of tiny tumors (∼0.048 mm^3^) (Fig. 8d).^49^ These studies demonstrate that second-level persistent afterglow requires atomic-scale proximity among PS/ECU/emitter units rather than nanoparticle-level colocalization, offering clear guidance for designing next-generation high-performance afterglow probes with superior brightness, persistence, and ROS generation.

*Expanding bioimaging horizons: from physiological barrier traversal to therapy-integrated afterglow imaging*. Beyond molecular innovation, afterglow probes are beginning to overcome physiological barriers that have long constrained conventional imaging. Compared with conventional fluorescent probes limited by poor barrier penetration and transient signals, Wang *et al.* harnessed macrophage-mediated transport to overcome the blood–brain barrier (BBB), enabling camouflaged afterglow nanoprobe (UCANPs@RAW) to achieve an exceptional SBR (∼258) in acute encephalitis *via* immune cell–guided delivery (Fig. 8f).^47^ This strategy surpasses passive diffusion of nanoparticles, offering a blueprint for neutrophil or exosome engineering and kinetic tuning aligned with disease dynamics. Meanwhile, Zhang *et al.* advanced interventional oncology by integrating arterial embolization therapy with real-time afterglow imaging.^48^ Their PA-gel undergoes an *in situ* phase transition to a calcium-crosslinked hydrogel, enhancing afterglow 11-fold and maintaining emission for 25 s after 2 s of laser pre-irradiation (Fig. 8g). Unlike short-lived fluorescent dyes, this design provides direct, durable visualization of vascular occlusion during therapy. Together, these contributions served to redefine afterglow imaging as an active theranostic platform rather than just a tool for passive readout. Such integrated control of delivery and transformation demonstrates how molecular design can potentially translate optical persistence into clinically relevant feedback for disease monitoring.

*Immunoassay revolution wherein ultra-sensitivity is achieved that extends beyond the autofluorescence limits*. One of the most transformative applications of afterglow probes lies with *in vitro* diagnostics, where they overcome the autofluorescence that limits conventional labels. Li *et al.* developed a lateral flow immunoassay (LFIA) based on afterglow nanoparticles (AGNPs) for antibody immunoassays.^46^ Based on the synergistic effect between SiPc and CUEM (cache unit-emitter molecular fusion), this technology eliminates autofluorescence and the light scattering interference of biological samples through the characteristics of afterglow signals, and further reduces background interference by virtue of time-resolved technology (Fig. 8e). Its ratio-based T/C line readout, which calculates the signal intensity ratio of the test line (T line) to the control line (C line), further reduced matrix interference by 89%. This approach enabled β-hCG detection at 0.34 mIU mL^−1^, approximately 15-fold below the clinical cutoff value (5 mIU mL^−1^), by effectively correcting for variations caused by sample matrices and operational conditions. Objectively, this design exemplifies a major advancement in point-of-care diagnostics, combining equipment-free operation with quantitative precision. By integrating long-lived afterglow emission and ratiometric analysis, it becomes possible to overcome the long-standing trade-off between simplicity and sensitivity, offering a versatile and clinically translatable platform for detecting trace biomarkers and emerging pathogens.

Together with advances in deep-tissue imaging and therapeutic integration, these diagnostic breakthroughs point to the rational design principles for future afterglow probes. (1) The first requirement is spatiotemporal precision: covalent confinement of PS, ECU, and emitter within single-molecule frameworks may enhance quantum yields. (2) The second is barrier-transcending delivery: cell-mimicking nanostructures, such as erythrocyte membrane–camouflaged ALNPs, may exploit physiological trafficking pathways for brain and fetal targeting while avoiding invasive procedures. (3) The third is visible theranostics: Integrating afterglow emission with stimuli-responsive drug release, such as protease-activatable chemotherapeutics, which could enable real-time monitoring of treatment efficacy during embolization therapy or immunotherapy.

### 2.5. Rubrene-based afterglow probes: superoxide activation mechanisms and dynamic tracking of acute kidney injury

**Fig. 9:**
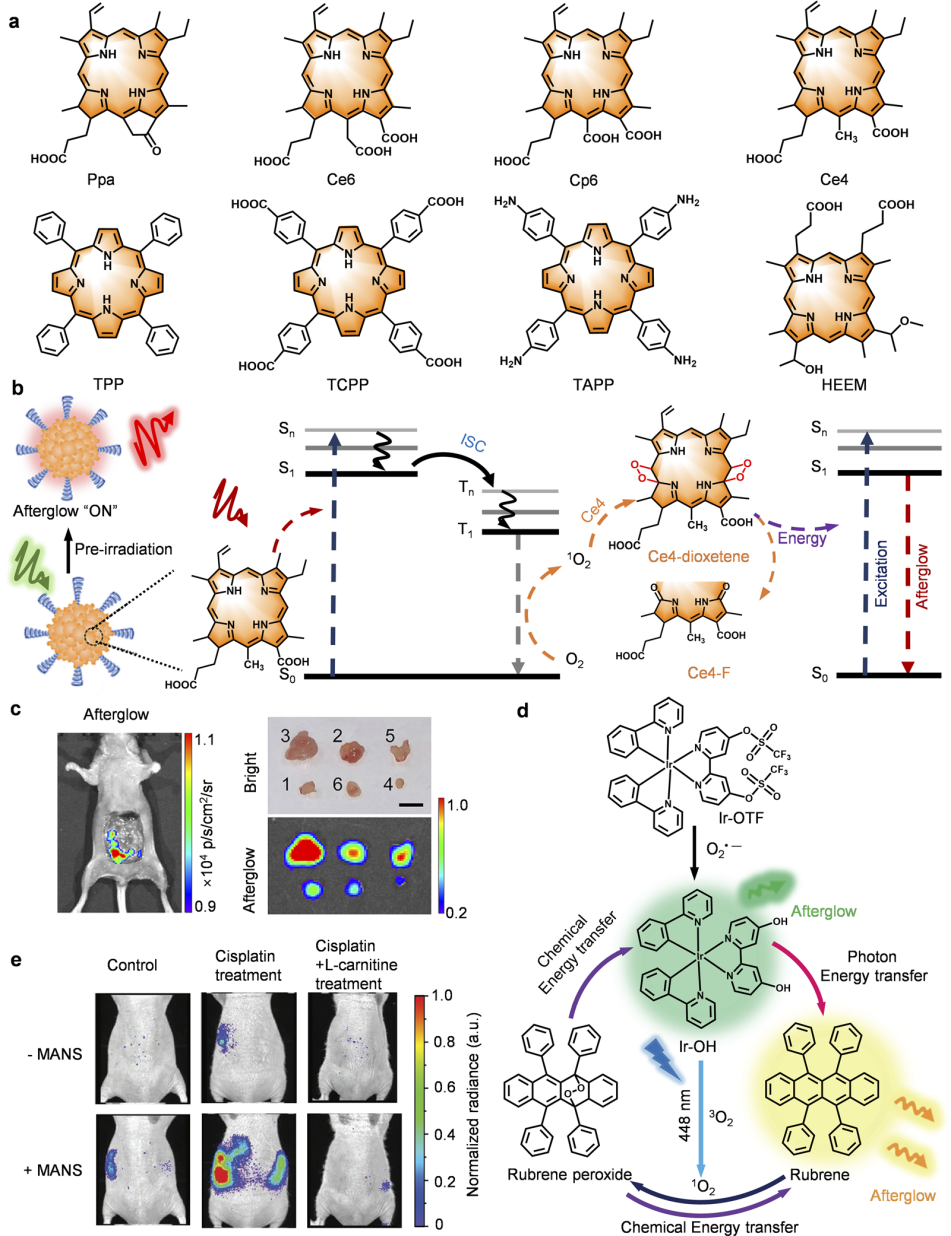
Afterglow probes based on porphyrin or rubrene for precise navigation of tiny metastatic tumors or dynamic tracking of acute kidney injury. (a) Chemical structure of porphyrin-based afterglow molecular substrate, and (b) afterglow luminescence mechanism and energy transfer mechanism. (c) Representative afterglow images of the tumor after injection of Ch-NPs. (d) Chemical structure and afterglow luminescence mechanism of rubrene-based molecular afterglow substrates. (e) Representative afterglow images of cisplatin-induced acute kidney injury (AKI) mice treated with PBS, cisplatin, or cisplatin with l-carnitine after injection of MANS. Panels b and c reprinted with permission from ref. 53. Copyright 2022 American Chemical Society. Panels d and e reprinted with permission from ref. 51. Copyright 2022 Wiley.

Traditional afterglow probes can effectively mitigate autofluorescence interference, but their typically complex structures make it difficult to integrate sensing functions while maintaining afterglow luminescence performance. In 2022, the introduction of the multifunctional components-incorporated afterglow nanosensor (MANS) by Kim *et al.* represents a deliberate step toward precisely engineered molecular cooperation.^51^ In contrast to conventional systems where emitters merely serve as passive energy acceptors, this design redefines rubrene, a polycyclic aromatic hydrocarbon long considered an inert emitter, as a dual-function component that simultaneously acts as both the afterglow substrate and light emitter. Through synergistic coupling with the superoxide-activated Ir-OTF/Ir–OH redox cycle, the system results in a well-ordered energy cascade that markedly enhances emission efficiency (Fig. 9d). As expected, in cisplatin-induced acute kidney injury (AKI) models (Fig. 9e), the probe (MANS) responded sensitively to renal O_2_˙^−^ elevation, producing a high-contrast afterglow (SBR ≈ 4) with virtually no background interference, a clear improvement over conventional luciferase reporters that suffer from tissue absorption and nonspecific luminescence. Upon l-carnitine pretreatment, which suppresses oxidative stress, the afterglow signal returned to baseline, providing direct biochemical validation of O_2_˙^−^ specificity. As such, this system demonstrates both mechanistic innovation and diagnostic reliability. By transforming a classical fluorescent molecule into an active afterglow generator through rational energy coupling, it achieves superior signal clarity, target specificity, and temporal stability. Moreover, its chemical activation mechanism offers better reproducibility and clinical adaptability than enzyme-dependent bioluminescence systems, underscoring its potential as a robust platform for noninvasive renal oxidative stress imaging.

Building on this breakthrough, the central contribution of MANS lies in uniting a stimuli-responsive molecular switch with an optimized energy-transfer cascade, thereby creating a transformative framework for background-free molecular imaging. Moving from concept toward clinical translation, several key challenges remain: (1) extending this cooperative design to pathological biomarkers beyond ROS, (2) addressing potential long-term cytotoxicity associated with heavy-metal components, and (3) advancing into the NIR-II window to achieve deep-tissue penetration. Overcoming these hurdles could enable MANS-inspired synergism to yield smart afterglow probes that map biochemical activity *in vivo* with a precision and continuity unattainable by conventional fluorescence.

### 2.6. Porphyrin derivative-based afterglow probes: near-infrared afterglow optimization and precision navigation of tiny metastatic tumors

Obscured by tissue autofluorescence and limited penetration depth, the persistent challenge of visualizing submillimeter metastatic tumors demands a fundamental departure from conventional fluorescence imaging. Porphyrin derivatives have emerged as promising afterglow luminophores, uniquely combining NIR emission (620–760 nm) with chemically tunable oxidative sites.^81,82^ In 2022, Ding's group established pyropheophorbide-a (Ppa) as a benchmark afterglow substrate (>760 nm emission, >1 h persistence) using β-sheet peptide self-assembly (FFG) to enforce rigid stacking (Fig. 9a).^52^ This structural order quenched fluorescence but amplified photoacoustic output, yielding a Ppa-FFGYSA conjugate with an unprecedented signal-to-background ratio (SBR) of 3,235, 42-fold higher than fluorescence. However, the compromise was slow kinetics, with tumor accumulation peaking 8 h post-injection, which limits intraoperative use. In contrast, Miao's chlorin-based nanoparticles (Ch-NPs) accelerated the response through carboxyl-functionalized molecular design (Fig. 9a).^53^ Unesterified carboxyl groups stabilized dioxetane intermediates and tripled singlet oxygen yield, while polymer encapsulation extended the afterglow half-life to 1.5 h and delivered a 26 : 1 SBR in deep tissue (Fig. 9b). Compared with Ding's submillimeter precision, Ch-NPs detected larger (3 mm^3^) metastases but at reduced spatial resolution (Fig. 9c).

### 2.7. Hemicyanine-based afterglow probes: modular design and dynamic monitoring of pathological markers

**Fig. 10:**
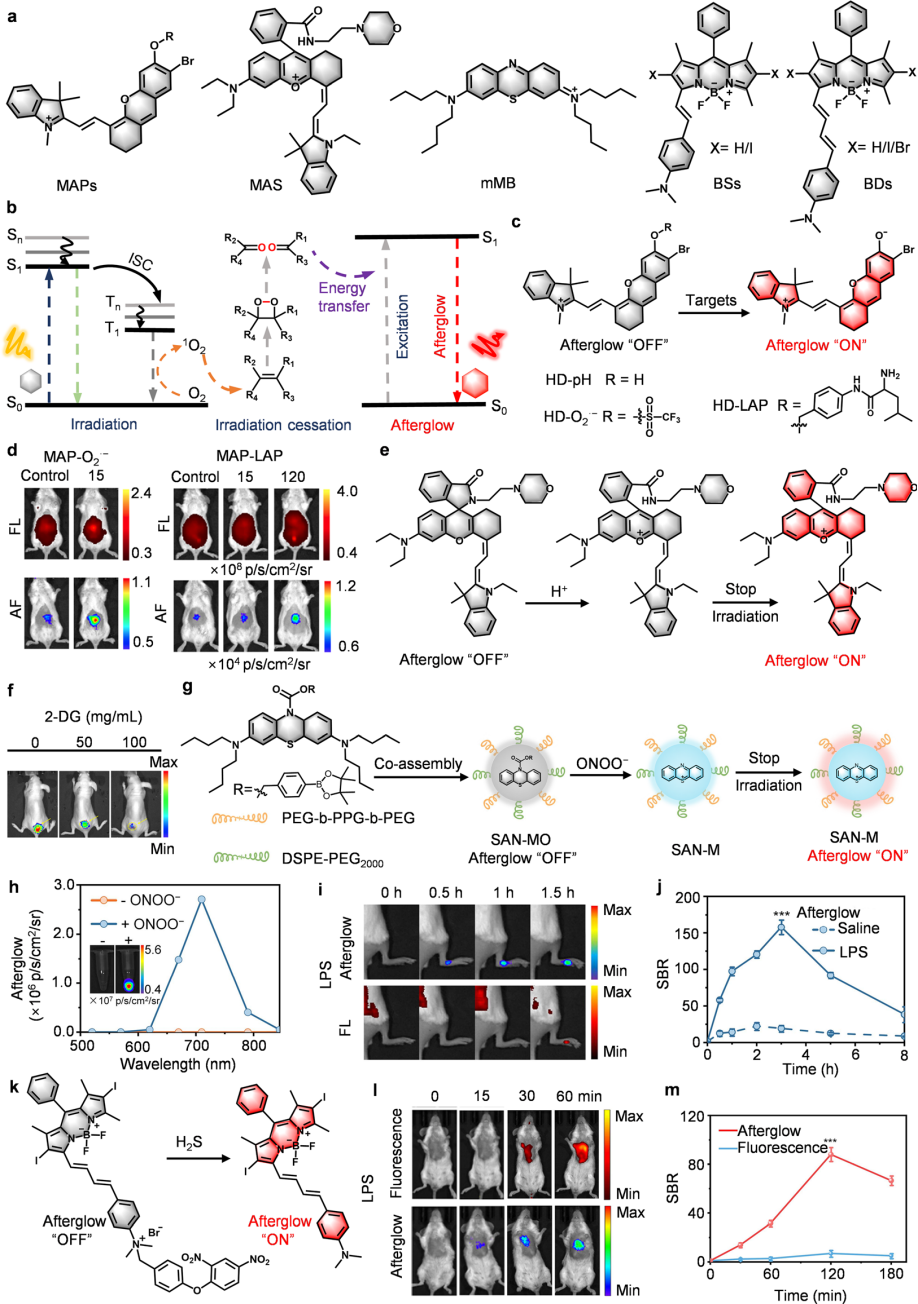
Afterglow probes based on hemicyanine or self-sustaining molecules for biomedical diagnosis. (a) Chemical structures of afterglow substrates, and (b) afterglow luminescence and energy transfer mechanisms. Afterglow activation mechanism of (c) MAPs and (e) MAS. (d) Representative afterglow and fluorescence images of the liver after injection of MAP-O_2_˙^−^ or MAP-LAP. (f) Representative afterglow images of subcutaneous doxorubicin-resistant human breast cancer (MCF-7 DR) xenograft-bearing mice tumors after injection of MAS-pH. (g) Schematic structure and afterglow activation mechanism of SAN-MO. (h) Afterglow luminescence in abscence or prescence of ONOO^−^. (i) Representative afterglow images of inflammatory sites after injection of SAN-MO, and (j) SBR. (k) Schematic structure and afterglow activation mechanism of BDIS NPs. Representative afterglow and fluorescence images of the lungs after injection of BDIS NPs, and (m) SBR. Panels b–d reprinted with permission from ref. 54. Copyright 2023 American Chemical Society. Panels e and f reprinted with permission from ref. 55. Copyright 2023 American Chemical Society. Panel g–j reprinted with permission from ref. 56. Copyright 2024 Wiley. Panel k-m reprinted with permission from ref. 58. Copyright 2025 Wiley.

The hemicyanine molecular scaffold represents a new concept in afterglow probe design through its “four-in-one” integration of a stimulus-responsive unit, an ^1^O_2_ generation unit, an ^1^O_2_ capture unit, and a luminescent unit.^54^ This modular activatable probe (MAP) architecture (Fig. 10a) constitutes a class of self-contained systems where intramolecular cascade photoreactions replace traditional multicomponent energy transfer. In HD-Br, the bromine's heavy-atom effect drives ISC efficiency to unprecedented levels, triggering a sequence where photoexcited ^1^O_2_ forms endoperoxides (EPOs) *via* cycloaddition. These metastable intermediates then release stored chemical energy intramolecularly, generating delayed luminescence without external sensitizers (Fig. 10b). This self-sustained energy capture system overcomes two key limitations of traditional afterglow probes: strict molecular ratios and slow diffusion. Meanwhile, their modular design enables tailored targeting, such as MAP-O_2_˙^−^ for superoxide detection and MAP-LAP for leucine aminopeptidase tracking (Fig. 10c). These advances have enabled the following key developments:

*Advancing afterglow imaging toward clinical early diagnosis*. Compared with conventional diagnostic methods such as serum biomarkers (ALT/AST) and histological staining (H&E, TUNEL), which only exhibit noticeable alterations after approximately 120 minutes, the MAP probe platform enables real-time, molecular-level detection of early hepatotoxic events. In the APAP-induced DIH model, MAP-O_2_˙^−^ captured the superoxide burst in liver tissue within just 15 minutes, while MAP-LAP visualized the upregulation of leucine aminopeptidase (LAP) as early as 120 minutes (Fig. 10d). This represents a temporal sensitivity far exceeding that of traditional pathological or serological indicators. Beyond temporal resolution, MAP probes exhibit a signal-to-background ratio (SBR) dozens of times higher than fluorescence imaging, completely free from interference from tissue autofluorescence. Such clarity allows submillimeter spatial mapping of oxidative stress and enzymatic activity during the early stages of liver injury, which provides an unprecedented precision for *in vivo* hepatotoxicity monitoring. Objectively, this performance highlights the methodological superiority and translational value of the MAP platform. Its modular design, achieved by functionalizing the semi-cyanine hydroxyl group with different responsive moieties, offers a universal and easily extendable strategy applicable to diverse pathological biomarkers. Consequently, MAP-based afterglow imaging not only bridges the gap between molecular sensing and clinical diagnostics but also establishes a versatile framework for early, noninvasive disease monitoring.

*Dynamic signal and metabolic sensing enhancements*. Building on the MAP framework, Song and his team have combined hemicyanine with rhodamine to develop a novel molecular afterglow scaffold (MAS) (Fig. 10a).^55^ Compared with conventional ICT-based pH-responsive afterglow probes, which often suffer from narrow response windows and low signal-to-noise ratios, MAS-pH introduces a rationally engineered rhodamine–indole spirolactam framework that realizes precise and synergistic control of both luminescence and photodynamic activity through pH-triggered ring opening (Fig. 10e). Under physiological pH (7.4), the closed-ring state minimizes background emission, while in the acidic tumor microenvironment (pH 6.1), ring opening extends π-conjugation, resulting in a 50.1-fold enhancement of afterglow, notably outperforming the modest 9.1-fold fluorescence increase of conventional systems. Additionally, a signal enhancement (ON/OFF) ratio of 44.9 (*i.e.*, afterglow intensity at pH 5.5 relative to pH 7.5), over an order of magnitude higher than that of traditional ICT-based probes, reflects not only superior responsiveness but also excellent quantitative fidelity for mapping subtle pH gradients. This expanded dynamic range allows MAS-pH to achieve more accurate tumor microenvironment imaging and reliable tracking of glycolytic metabolism (Fig. 10f), demonstrating its clear practical advantage and translational potential over earlier pH-sensitive afterglow designs.

These advances highlight the shift from conceptual systems toward clinically relevant probe platforms. However, to enable afterglow theranostics, future breakthroughs in designing hemicyanine-based afterglow probes requires three transformative upgrades: (1) cascade intelligence: we suggest the replacement of single-path activation with multivariate logic gates and dynamically reversible switches responsive to gradients of enzyme/pH/redox. Such systems could autonomously discriminate inflamed tissue *versus* malignant tissue. (2) Highly efficient energy transfer: maximize exciton utilization through triple-strategy engineering: heavy-atom optimization (*e.g.*, I > Br > Cl), stability-tuning of EPO intermediates *via* spirocyclic rigidification, and donor–acceptor alignment (<0.3 eV gap) to minimize thermal dissipation. (3) Multimodal integration for clinical application: develop drug-loaded afterglow probes for drug release, self-powered photodynamic hybrids (afterglow → ^1^O_2_ → apoptosis), and afterglow-photoacoustic bimodal agents for cross-validated tumor margin delineation.

### 2.8. Self-sustaining afterglow molecules: rational design from methylene blue to BODIPY and biomarker-activatable imaging

The development of molecular afterglow probes has moved beyond multi-component complexity *via* modular functional integration. The self-sustaining molecular afterglow imaging probe (SAN-M) developed by Miao's team represents a significant advancement in the field (Fig. 10a).^56,83,84^ By engineering phenothiazine ring modifications and dibutylamino alkyl chains, SAN-M achieves trifunctional synergy: strong ^1^O_2_ generation (*ε* = 2.1 × 10^5^ M^−1^ cm^−1^ at 675 nm), high-energy dioxetane intermediate, and NIR afterglow emission (710 nm) *via* intramolecular energy transfer.

A current research frontier is centered around the so-called self-sustaining afterglow strategy. This strategy is rooted in a fundamentally new energy-harvesting principle, in which photoexcited methylene blue derivative (mMB, the core molecule of SAN-M) simultaneously functions as the photosensitizer, the energy reservoir, and the emitter within a single molecule. The spontaneous decomposition of the dioxetane intermediate releases chemical energy directly into the emissive unit, thus bypassing intermolecular diffusion, which overcomes a critical limitation in earlier multi-component systems. Such an intramolecular confinement achieves three advantages, including ultrahigh quantum yield (*Φ*_at_ ≥ 0.18), hypoxia-insensitive afterglow, as well as biomarker-specific adaptations. The translational potential of this design becomes clear in SAN-MO, an activatable derivative equipped with phenylboronic ester groups for ONOO^−^ responsiveness (Fig. 10g, h). In LPS-induced inflammation models, SAN-MO detected peroxynitrite surges within 30 minutes and achieved an exceptional SBR, outperforming fluorescent probes that barely detect signals within the same time window (Fig. 10i and j). This spatiotemporal precision arises from a tripartite synergy: structural tunability of the methylene blue scaffold, autonomous intramolecular energy conversion that eliminates the need for external sensitizers, and analyte-specific activation that ensures precise target engagement.

Following the methylene blue derived systems, the design concept of self-sustaining afterglow molecules (SAMs) has been further explored and expanded within the classic fluorescent framework of boron dipyrromethene (BODIPY) derivatives (Fig. 10a).^58^ To overcome the inherent complexity of optimizing multi-component systems and explore rational design principles from the molecular level, Miao *et al.* recently reported a self-sustaining, modular afterglow scaffold based on BODIPY derivatives. The core molecular engineering strategy involves: introducing halogen atoms (iodine or bromine) into the BODIPY core to leverage heavy atom effects, promoting ISC and thereby significantly enhancing the generation efficiency of ^1^O_2_; and extending the conjugation *via* terminal ethylene bonds. This design not only red-shifts absorption and emission spectra but, more crucially, increases the chemical reactivity of the molecular skeleton toward ^1^O_2_, thereby facilitating its oxidation into high-energy peroxide intermediates. Within this system, a single BDI molecule integrates three functions: photosensitizer, chemical reaction substrate, and luminescent unit, achieving self-sustained near-infrared afterglow emission peaking at 780 nm. This research, through systematic investigation of the structure–property relationship, provides clear design guidelines for the rational bottom-up construction of SAMs.

Notably, the inherent structural tunability and ease of derivatization of the BODIPY skeleton make it an ideal platform for constructing high-performance activatable probes. Based on these design principles, researchers have covalently attached biomarker-responsive units (*e.g.*, units responsive to H_2_S) to the terminal *N*,*N*-dimethylaniline group of BDI, successfully constructing an “off-on” activatable afterglow probe (BDIS-NPs) (Fig. 10k). The probe achieved simultaneous activation of fluorescence, afterglow, and ^1^O_2_ generation in response to H_2_S. Leveraging the unique advantage of afterglow imaging in eliminating autofluorescence, BDIS-NPs enabled ultra-early monitoring within 15 minutes in LPS-induced acute lung injury models (Fig. 10l). In MK-801-induced schizophrenia mouse models, the system enabled the visualization of abnormal H_2_S accumulation in the mouse brain with high SBR—performance that is difficult to achieve with conventional fluorescence imaging (Fig. 10m). This work not only demonstrates the feasibility of BODIPY-based SAMs for precise activation imaging in complex biological environments, but also highlights the significant potential of integrating rational molecular design with disease-specific responses.

The challenge now lies in transforming these exquisite molecular machines into clinically deployable precision diagnostics. We expect that the following improvements could accelerate the clinical applications. (1) Enhancing dynamic reversibility is critical. Current probes operate irreversibly, while spirolactam-like switches could enable the real-time monitoring of fluctuating biomarkers. (2) Clinically translatable targeted strategies are needed: engineering zwitterionic surface modifications could reduce immune clearance while maintaining tumor permeability, thereby accelerating adoption in clinical precision diagnostics.

## 3. Ultrasound-activated afterglow probes: deep penetration and immune microenvironment monitoring

**Fig. 11:**
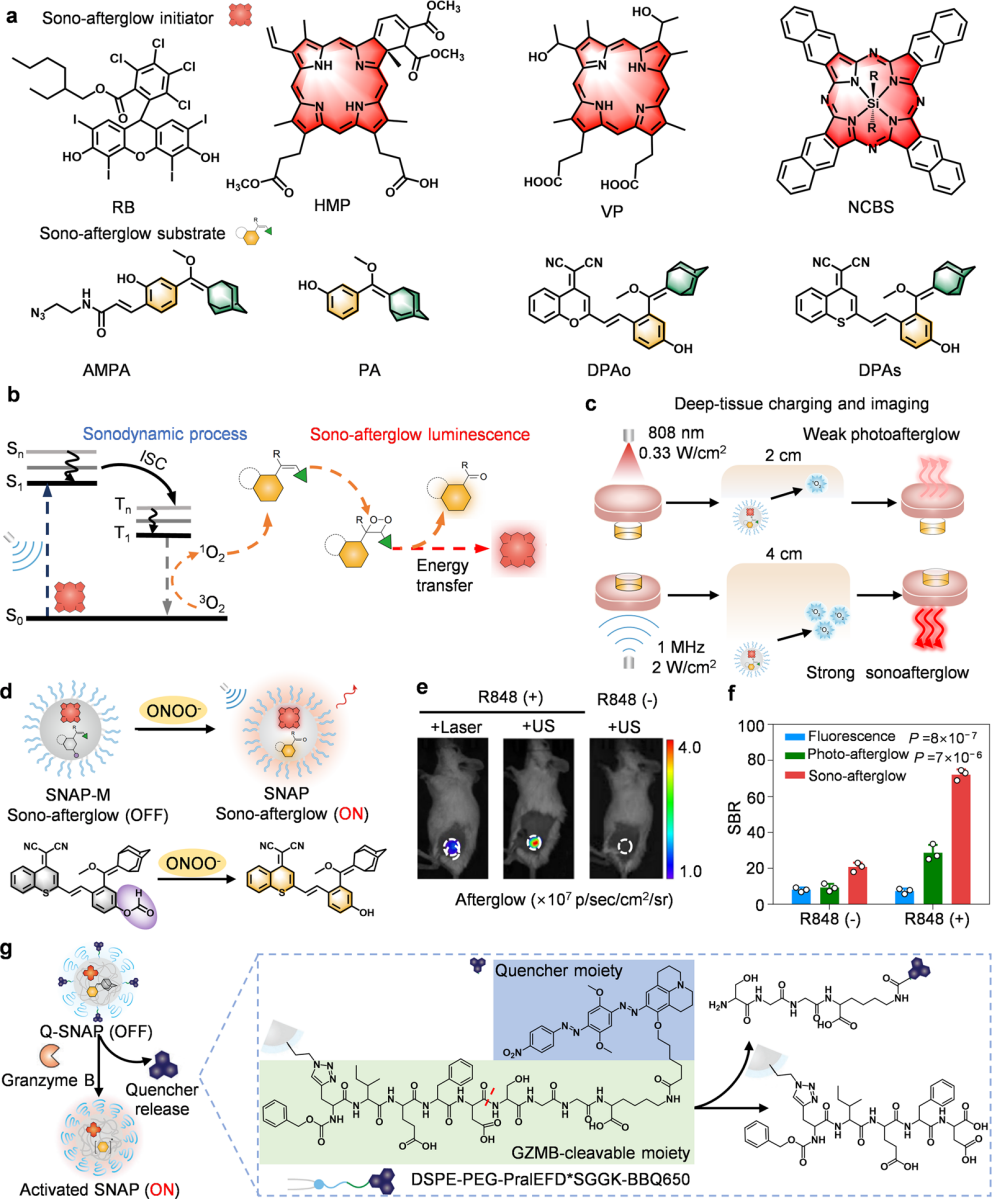
Ultrasound-activated afterglow probes for tumor-specific imaging. (a) Chemical structures of afterglow substrates and initiators. (b) Afterglow luminescence and energy transfer mechanisms of SNAPs. (c) Schematic diagram of tissue penetration ability of photo-afterglow and sono-afterglow. (d) Schematic diagrams of structures and afterglow activation mechanism of SNAP-M. (e) Representative afterglow images of subcutaneous murine breast cancer (4T1) tumors in Balb/c mice after injection of SMAP-M, and (f) SBR. (g) Schematic diagrams of structures and afterglow activation mechanism of Q-SNAP. Panels b–f reprinted with permission from ref. 30. Copyright 2022 Springer Nature. Panel g reprinted with permission from ref. 60. Copyright 2023 Wiley.

With photo-activated afterglow imaging, although tissue autofluorescence has been eliminated during signal acquisition, the penetration depth (<2 cm) into the tissue is still limited. However, we anticipate that ultrasound (US)-activated probes can be used to overcome optical diffusion limits.^85–87^ Pu's work on Sono-Afterglow Probes (SNAPs) pioneered this frontier by engineering acoustic sensitizers NCBS that generate cytotoxic ^1^O_2_ under US, triggering dioxetane formation in afterglow substrates such as DPAs (Fig. 11a).^30,88,89^ The intramolecular energy recycling mechanism uses energy from dioxetane decomposition to both red-shift emission and reactivate the sensitizer (Fig. 11b). This process enables tissue penetration up to 4 cm, breaking the 1–2 cm limit of fluorescence and photo-afterglow (Fig. 11c).

*Precision immune-monitoring: from macrophages to T-cell activity*. SNAP-M, responsive to ONOO^−^ in M1 macrophages, tracked polarization shifts within 36 hours and achieved a landmark SBR (∼58.2) in R848-induced inflammation (Fig. 11d–f). Extending this principle, Q-SNAP monitored T-cell activity, where granzyme B (GZMB)-mediated peptide cleavage released persistent afterglow that correlated linearly with cytotoxic T-cell infiltration (Fig. 11g).^60^ By combining centimeter-level depth penetration with cell-type specificity, SNAPs effectively create an “immune activity tomography” that surpasses the superficial snapshots offered by fluorescence imaging.

**Fig. 12:**
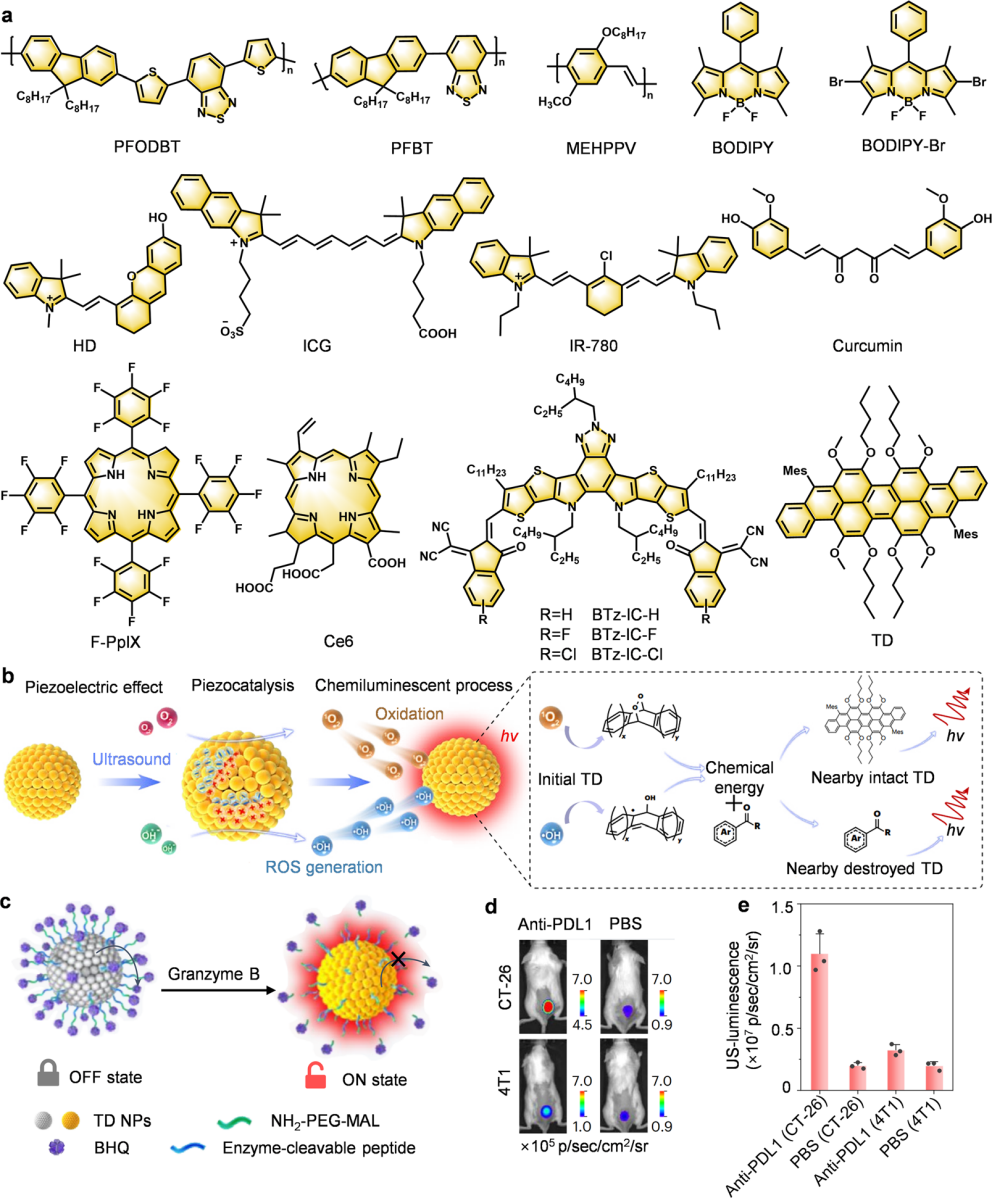
Ultrasound-activated afterglow probes for tumor-specific imaging. Schematic diagram showing (a) chemical structures of afterglow substrates, and (b) afterglow luminescence and energy transfer mechanisms of TD NPs. (c) Schematic diagrams of afterglow activation mechanism, and (d) representative afterglow images and (e) representative afterglow luiminescence intensities of subcutaneous murine colon cancer (CT-26) tumors and subcutaneous murine breast cancer (4T1) tumors in female Balb/c mice after injection of TD-Grz-BHQ. Panels b–e reprinted with permission from ref. 31. Copyright 2024 Springer Nature.

*Piezo-chemiluminescence synergy: a major leap in energy harvesting*. Following the important progress made by Pu's team in the field of ultrasound-activated organic afterglow probes, ultrasound-induced afterglow luminescence imaging continues to receive attention due to its deep tissue penetration. However, how to further optimize the ultrasound energy conversion efficiency, enhance the luminescence intensity, and expand the multifunctional applications of the probes are still the key challenges in this field.^28,90^ In this context, in 2024, Tan's group introduced piezoelectric nanoparticles (TD NPs, trianthracene derivative-based nanoparticles) as a novel strategy for energy harvesting (Fig. 12a).^31,91,92^ The technique achieves afterglow signal amplification through two-step energy conversion: first, piezoelectric properties of the nanoparticles are utilized to convert mechanical vibrations into chemical energy, and subsequently an optical signal is generated through a chemiluminescence process (Fig. 12b). This dual cascade mitigates the “energy leak” of conventional SNAPs, sustaining luminescence beyond US exposure while achieving 2.2 cm penetration. A GZMB-responsive probe (TD-GRz-BHQ) further enabled real-time discrimination of immunologically “hot” *versus* “cold” tumors, offering dynamic assessment of therapeutic efficacy (Fig. 12c–e).

Despite transformative progress, several hurdles remain: (1) energy conversion efficiency remains modest (<15% *vs.* ∼95% in piezoelectric). In order to solve this issue, we suggest that a combinatorial screening of π-extended sonosensitizers such as bacteriochlorin analogs paired with dioxetanes-bearing electron donor groups may further boost the yields of ^1^O_2_. (2) Limited luminescence intensity restricts sensitivity. As such, we propose hybridizing afterglow substrates with quantum-confined materials, like CsPbBr_3_ nanocrystals, to exploit Förster resonance energy transfer (FRET) amplification. (3) Achieving synergistic theranostics remains challenging. Existing sono-afterglow probes primarily excel at deep-tissue imaging or single-type immune monitoring,but lack synergy between imaging and therapeutic effects. To solve this, embedding afterglow probes within stimuli-responsive hydrogels could enable simultaneous deep-tissue immune activity tomography and on-demand immunotherapy, integrating diagnostic and therapeutic functions into a single system.

Since the ultrasound technique has already been used for some applications, we envision the development of “autonomous molecular sonars” based on the following approach: first, bioorthogonal approaches: strain-promoted click reactions for *in vivo* probe assembly at target sites. Second, dynamic feedback: encoding protease-cleavable spacers can modulate afterglow duration with immune activity. Third, clinical pilot: validating the application of afterglow probes suitable for human clinical settings in monitoring immune activation. We believe that merging enzyme-directed afterglow amplification will unlock sub-centimeter resolution, and ultimately facilitate deep-tissue immune monitoring.

## 4. Radio-activated afterglow probes: synergistic deep tumor imaging and radio-dynamic treatment

**Fig. 13:**
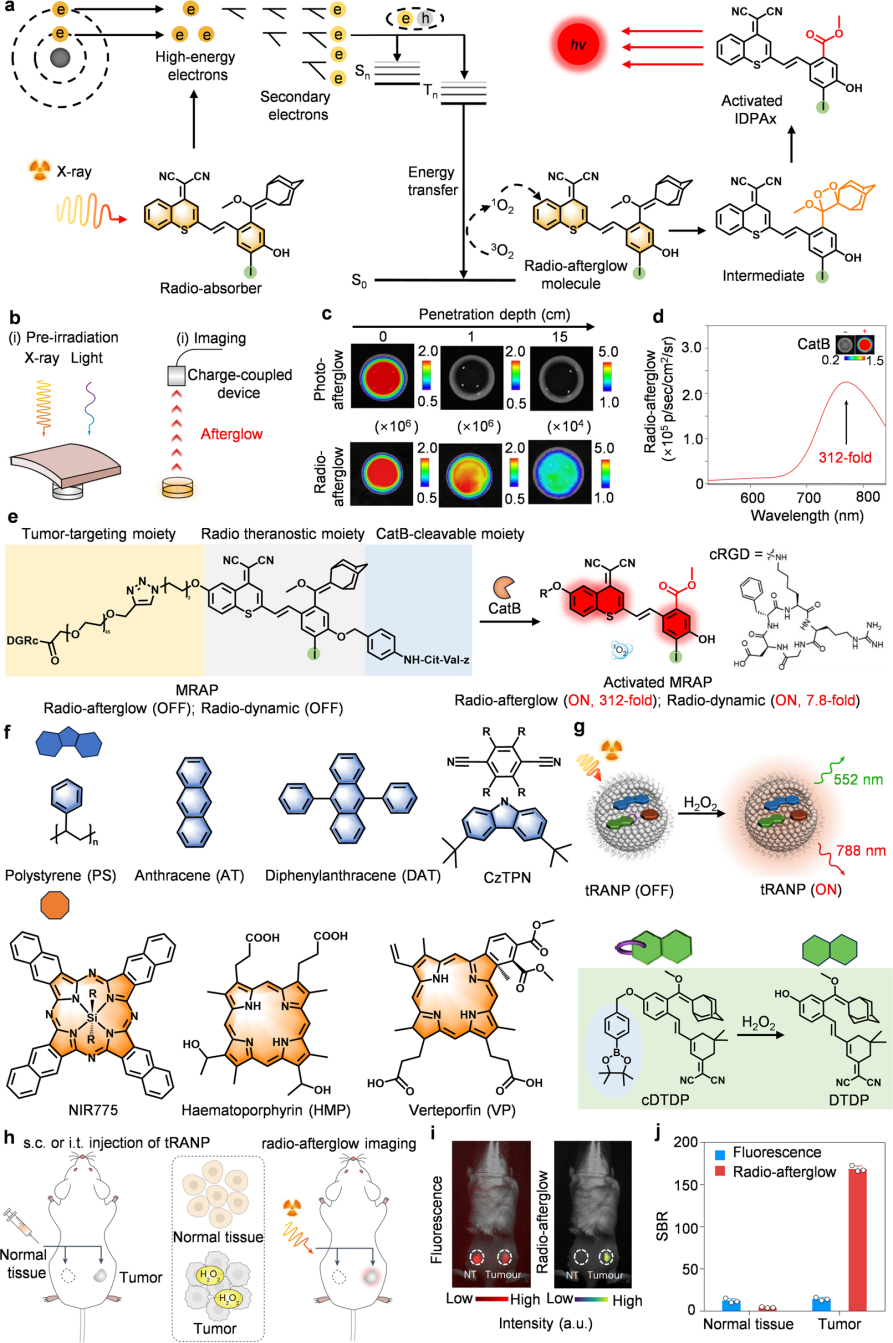
Radio-activated afterglow probes for biomedical imaging. (a) Radio-activated afterglow luminescence mechanism and energy transfer mechanism. (b) Schematic diagram of tissue penetration ability of photo-afterglow and radio-afterglow, and (c) representative afterglow images. (e) Schematic diagram of the structure and afterglow activation mechanism of MRAP, and (d) SBR. (f) Schematic diagram of the structure, and (g) afterglow activation mechanism of tRANP. (h) Schematic diagrams of radio-afterglow-specific imaging. (i) Representative radio-afterglow and fluorescence images of 4T1 tumor-bearing mice after injection of Q-SNAP, and (j) SBR. Panels a–e reprinted with permission from ref. 32. Copyright 2023 Springer Nature. Panels f–j reprinted with permission from ref. 33. Copyright 2024 Springer Nature.

Non-invasive deep-tissue imaging and therapy have long been constrained by the penetration limits of conventional stimuli like light or ultrasound. In contrast, X-rays, with their unparalleled tissue-penetrating capability, emerge as a transformative alternative.^93–97^ However, their potential remains underexploited due to inefficient energy conversion and poor spatiotemporal control. Herein, we propose that radio-activated afterglow probes represent not merely an improvement, but also practical tools for precision oncology. Unlike light-activated systems that fail beyond a depth of 3 cm, these probes exploit Compton scattering and photoelectric effects to generate high-energy electrons, which in turn produce triplet excitons for sustained afterglow (Fig. 13a). From the perspectives of mechanism, we can find that the afterglow luminescence lifetime depends mainly on the generation of dioxetane intermediates from ^1^O_2_-oxidized molecules and the decomposition rate of the intermediates, rather than on X-ray excitation. This is because the central role of X-rays is to achieve deep energy delivery to trigger ^1^O_2_ production, whereas the afterglow lifetime is dominated by the chemical structure of the molecule. As such, optimization of the afterglow lifetime can be achieved by chemically modulating the stability of the dioxetane, thus providing a remarkable prospect for the development of organic radio-afterglow luminescent probes.

*Molecular innovation for clinical translation*. Recent breakthroughs by Pu *et al.* epitomize this strategy.^32^ Their IDPA_Su_ probe, a covalent fusion of X-ray sensitizers and afterglow substrates, achieves unprecedented 15 cm penetration of tissue, eclipsing optical methods 5-fold and enabling imaging of deep-seated organs like the pancreas (Fig. 13b and c). More strikingly, their enzyme-activatable MRAP probe amplified the afterglow signals 312-fold while boosting ^1^O_2_ production 7.8-fold (Fig. 13d and e). This dual signal-and-therapeutic amplification achieved selectivity unattainable by pH/ROS-responsive systems, and redefines theranostic precision. Conventional probes suffer from false positives, while MRAP's Cathepsin B (CatB) specificity slashes background noise, yielding a 3-fold improvement over prior tumor-to-background ratios.

Beyond imaging there exists an opportunity for synergistic radiodynamic therapy. Earlier radio-afterglow materials faced critical limitations: low X-ray absorption and suboptimal ^1^O_2_ conversion rates (<15%), crippling their therapeutic efficacy. Compared with conventional radioluminescent systems that suffer from limited penetration, nonspecific activation, and high radiation burden, Pu's cascading energy conversion probe (RANP) represents a substantial advance in both design logic and translational potential.^33,98^ By integrating a heavy-atom–doped radioabsorber,^99^ a NIR775 radiosensitizer, and an H_2_O_2_-responsive afterglow substrate into a single nanoplatform, RANP achieves a synergistic enhancement in imaging depth (up to 15 cm), signal release (40-fold), and safety (radiation dose ≤ 1 Gy *versus* the conventional 5 Gy) (Fig. 13f and g). Furthermore, the phenylboronate-based caged structure exemplifies the molecular precision of this design: SBR > 169 with rapid metabolic clearance (<4 h) and low radiotoxicity (Fig. 13h–j). Objectively, this system demonstrates how rational cascade integration can reconcile the traditional trade-offs of penetration depth, biosafety, and specificity. While clinical translation will still depend on long-term dosimetry and scalable synthesis, RANP defines a new design paradigm in which programmable energy transfer and bioresponsive activation jointly drive safe, deep-tissue afterglow imaging, marking a significant stride toward human-applicable radiotheranostics.

Although some progress has been made, we must remain vigilant about the challenges ahead. For example, though dioxetane intermediates empower lifetime tuning, their *in vivo* metabolic stability remains suboptimal; Heavy-atom sensitizers, though efficient, risk long-term toxicity. As such, we propose that these three research directions should be pursed: (1) lanthanide-based X-ray capture, which could contribute to deep tissue penetration enabling afterglow probes to function at depths up to 25 cm. (2) Bioorthogonal catalysis which might push activation ratios beyond 100-fold while avoiding metal toxicity. (3) Dynamic modulation of X-ray doses with real-time afterglow feedback which will enable individual adaptive therapy. It is conceivable, that radio-afterglow probes, optimized for blood–brain barrier penetration, could monitor neurotransmitter dynamics in the brain, which would be game changing in the diagnosis and treatment of neural diseases like Parkinson's or epilepsy.

## 5. Comparison and outlook of photo-activated, sono-activated, and radio-activated afterglow probes

As mentioned earlier, organic afterglow probes can be primarily categorized into three types depending on their external energy source: photo-activated, sono-activated, and radio-activated. This section aims to systematically summarize and compare these three excitation modes, while outlining their future development prospects.

### 5.1. Discussion of excitation mechanisms and characteristics

Photo-activated afterglow probes have been the most extensively studied among such systems, with their mechanisms clearly elucidated and their structures highly diverse. They have found broad applications ranging from *in vitro* detection to *in vivo* superficial-tissue imaging. However, due to scattering and absorption of light in biological tissues, their tissue penetration depth is typically limited to 1–2 cm, thereby limiting their application in deep tissue imaging.

Sono-activated afterglow probes utilize ultrasound as the excitation source. Their core mechanism involves converting mechanical energy into chemical energy, either through sonosensitizers or the piezoelectric effect, thereby triggering afterglow luminescence. This strategy has elevated imaging depth to the centimeter scale (*e.g.*, 4 cm) and enabled monitoring of deep-tissue immune microenvironments. However, the energy conversion efficiency and luminescence intensity of this technology still require further improvement.

Radio-activated afterglow probes leverage the exceptional tissue penetration capabilities of X-rays. Their mechanism involves the generation of high-energy electrons through effects such as Compton scattering, exciting sensitizers to produce ^1^O_2_, thereby triggering afterglow emission. This class of probes fundamentally overcomes the depth limitations of optical imaging, offering a revolutionary tool for high SBR optical imaging of whole body or deep-seated organs. Currently, the primary challenges lie in further improving X-ray energy conversion efficiency and optimizing the biosafety profile of the probes.

### 5.2. Performance comparison and application prospects

The distinct characteristics of afterglow probes activated by these three different excitation modes determine their unique application scenarios and development directions. Table 2 provides a systematic comparison of their key performance parameters, advantages, limitations, and prospects.

**Table 2:** Key differences between photoafterglow, sonoafterglow, and radioafterglow

Property	Photoafterglow	Sonoafterglow	Radioafterglow
Excitation source	Visible/NIR light	Ultrasound	X-ray
Core mechanism	Photosensitizer absorbs photons and generates ^1^O_2_*via* ISC	Sonosensitizer generates ^1^O_2_*via* ISC; piezoelectric effect converting mechanical energy into chemical energy	Absorption of high-energy photons/particles produces secondary electrons, activating sensitizers to generate ^1^O_2_
Tissue penetration depth	Limited (typically < 2 cm)	Deep (up to 4 cm)	Ultra-deep (experimental verification reached 15 cm)
	Restricted by light scattering and absorption	Low ultrasound attenuation in soft tissues	Enabling whole-body or deep organs
Imaging SBR	High	High	Very high
	Signal acquisition after excitation avoids autofluorescence	Absence of optical background, though sonoluminescence conversion efficiency affects sensitivity	Minimal optical background in deep tissue
Key advantages	High spatiotemporal resolution	Strong deep-tissue penetration	Exceptional penetration depth
	Highly flexible molecular design	Excellent biosafety and clinical compatibility	Applicable to lesions beyond reach of conventional optics
Main biomedical applications	Intraoperative navigation; tumor imaging; *in vitro* diagnostics; biomarker detection	Deep-tissue tumor imaging; monitoring of immunotherapy; synergistic sonodynamic therapy	Whole-body deep-tissue imaging; guidance and monitoring of radiotherapy/radiodyamic therapy
Current challenges	Limited tissue penetration depth; susceptibility to oxygen/water quenching in biological environments	Low acoustic-to-optical energy conversion efficiency	Weak X-ray absorption by organic materials
		Limited variety of efficient sonosensitizers/piezoelectric materials	Energy conversion efficiency requires optimization
		Imaging sensitivity and spatiotemporal resolution need improvement	Radiation dose control and long-term biosafety
			Clinical translation involves radiation safety regulations
Future directions	Develop NIR-II emitting materials	Develop novel efficient sonosensitizers and piezoelectric materials	Develop highly efficient and low-toxicity afterglow probes
	Construct multi-responsive intelligent systems	Explore synergistic effects of ultrasound and immunotherapy	Promote integration of miniaturized, portable radiation sources with imaging devices
	Improve brightness and lifetime	Optimize probe sensitivity and specificity	

In summary, photo-activated, sono-activated, and radio-activated afterglow imaging technologies are advancing along their distinct developmental paths. By gaining a deeper understanding of their intrinsic mechanisms and addressing their respective key challenges, these three technologies hold great promise to synergize future precision medical systems. We anticipate that these systems will provide more powerful and versatile non-invasive visualization tools across all levels, from fundamental research to clinical diagnosis and treatment.

## 6. Conclusions

As an emerging diagnostic method, deep afterglow imaging exhibits considerable potential for biomedical research, disease diagnosis and, in the case of certain systems, treatment. We believe that the design strategies and research progress involving afterglow imaging probes with deep tissue excitation will result in agents that will support several future applications. Possible approaches to realizing this promise could involve the following:

(1) Chemical modifications and nanoengineering strategies can be integrated to improve the properties of current afterglow probes. Using chemical modifications (*e.g.* donor–acceptor structure, extension of conjugation, introduction of functional groups) and nanoengineering strategies (*e.g.* self-assembly, targeting group modifications), probes with long afterglow lifetime, near-infrared emission, high brightness and biomarker targeting will be possible, enhancing their practicality.

(2) Improving the core mechanism of afterglow luminescence. The basis for afterglow is the redox cascade reaction between the excited state active substance and the probe. External energy excites the probe molecule to generate highly reactive intermediates (*e.g.*, ^1^O_2_), which oxidize specific chemical bonds in the molecular skeleton to form dioxetane intermediates. These intermediates release chemical energy through rupture of O–O bond, leading to an electron transition to the excited state which subsequently results in afterglow luminescence through chemically induced electron-excited luminescence (CIEEL) or chemiluminescence resonance energy transfer (CRET). The initial energy conversion paths differ for different excitation sources (light, ultrasound, X-rays): photo-excitation relies on the intersystem crossing (ISC) of the photosensitizer to generate ^1^O_2_; Ultrasonic excitation generates ^1^O_2_ through ISC of the sonosensitizers or by converting mechanical fluctuations into chemical energy; X-ray excitation generates ^1^O_2_ through the activation of sensitizers by secondary electrons, which are produced *via* ionization of surrounding media by high-energy primary electrons. Although the three excitation sources have different energy conversion paths, all of them generate energetic intermediates (dioxane intermediates) by ^1^O_2_ oxidizing the afterglow substrates. These intermediate releases energy during spontaneous decomposition to realize afterglow luminescence.

(3) Multi-scenario adaptability for *in vivo* diagnostics and *in vitro* diagnostics. With high SBR and real-time monitoring capabilities, afterglow imaging molecular probes exhibit enhanced practicality for both *in vivo* diagnostics (*e.g.*, tumor/lymph node imaging, drug-induced liver injury monitoring) and *in vitro* diagnostics (*e.g.*, cancer exosome detection, pregnancy diagnosis).

(4) Integration of afterglow imaging probes with other medical imaging techniques can enhance clinical practicality. The combination of afterglow imaging techniques with magnetic resonance imaging (MRI), photoacoustic imaging, radiation therapy and other multimodal technologies will result in practical theranostic applications.

Although considerable fundamental progress has been made toward organic afterglow imaging probes, there are still many important goals that remain to be realized.

(1) Signal attenuation and background interference issues in deep tissue imaging. Currently, the emission wavelengths of most probes remain concentrated in NIR-I (700–900 nm), where signals experience significant attenuation due to scattering and absorption when penetrating deep tissues (>5 cm). Although some probes have extended their emission into NIR-II, they still face challenges such as low excitation efficiency and insufficient luminescence quantum yield. Additionally, metabolic byproducts, hemoglobin, and water within biological organisms can generate non-specific background signals, and such background interference is particularly pronounced during low-concentration biomarker detection or imaging of minute lesions. Future efforts are needed to further optimize the spectral properties of probes and enhance their resistance to fluorescence quenching.

(2) Biocompatibility and *in vivo* metabolic safety of probes. In current probe designs, the introduction of heavy atoms (*e.g.*, iodine, bromine) helps enhance intersystem crossing efficiency and X-ray absorption capacity, but their long-term retention in the body may lead to cumulative toxicity. Furthermore, polymer carriers (*e.g.*, PPV derivatives, PEG-modified materials) exhibit poor biodegradability, readily accumulating in organs and inducing inflammatory responses. Additionally, the metabolic pathways of products released by activated probes after response remain unclear, and certain high-energy chemical intermediates (*e.g.*, dioxetane derivatives) could potentially exhibit biological toxicity. These issues collectively limit the clinical translation prospects of such probes. Future efforts should focus on developing biodegradable molecular frameworks, screening low-toxicity alternatives, and systematically optimizing metabolic pathways to address these challenges.

(3) Insufficient energy transfer efficiency between excitation sources and probes. Photoactivated probes are limited by tissue penetration depth (typically <2 cm), making them unsuitable for imaging deep-seated tumors. Ultrasound-activated probes generally exhibit low energy conversion efficiency (typically <15%), with significant energy loss occurring during the transformation from mechanical to chemical energy. X-ray-activated probes possess strong penetration capabilities but face a trade-off between radiation dose control and energy utilization efficiency: high-dose radiation may damage normal tissue, while low doses struggle to trigger sufficient ^1^O_2_ generation. Additionally, existing excitation equipment (such as clinical ultrasound machines and X-ray machines) lack sufficient compatibility with afterglow probes, and the lack of dedicated signal acquisition and analysis systems further limits the practical application of this technology.

(4) Optimization of activatable probes specificity and response kinetics. Currently, most activatable probes rely on a single biomarker (*e.g.*, H_2_O_2_, CatB, pH) for triggering. However, the microenvironments of tumors and inflammation often involve coordinated changes in multiple biomarkers, making a single-response mode prone to generating false-positive signals. Furthermore, the response kinetics of some probes are relatively slow, hindering real-time capture of dynamic biomarker changes, while overly rapid responses may cause premature signal decay. Consequently, future development should focus on developing probe architectures with multimodal response capabilities (*e.g.*, dual pH/enzyme responses, ROS/hypoxia synergistic responses) and tunable kinetic properties.

Addressing these challenges is expected to lead to the development of more practical, precise and efficient imaging probes. At the same time, through interdisciplinary collaboration afterglow imaging is expected to advance from the laboratory to the clinic and evolve as a key tool for precision medicine.

## Conflicts of interest

There are no conflicts to declare.

## Abbreviations

AIE: Aggregation-induced emission
AEE: Adamantylidene enol ethers
ATP: Adenosine triphosphate
ALT: Alanine transaminase
AST: Aspartate transaminase
AKI: Acute kidney injury
BHQ-2: Black hold quencher 2
CRET: Chemiluminescence resonance energy transfer
CIEEL: Chemically induced electron-excited luminescence
DA: 2,3-Dimethylmaleic anhydride
DAMPs: Damage-associated molecular patterns
5-DFUR: 5′-Deoxy-5-fluorouridine
ECU: Energy cache unit
ESIPT: Excited-state intramolecular proton transfer
ΔEST: Singlet-triplet energy gap
ET: Energy transfer
FRET: Fluorescence resonance energy transfer
FDA: Food and Drug Administration
FFG: FFGY SAYPDSVPMMS
GZMB: Granzyme B
GSH: Glutathione
H_2_O_2_: Hydrogen peroxide
H_2_S: Hydrogen sulfide
H&E: Hematoxylin and eosin
Hcy: Homocysteine
ICD: Immunogenic cell death
ICG: Indocyanine green
ICT: Intramolecular charge transfer
ISC: Intersystem crossing
IL-6: Interleukin-6
LFIA: Lateral flow immunoassay
LAP: Leucine aminopeptidase
MRI: Magnetic resonance imaging
ROS: Reactive oxygen species
NIR: Near-infrared
NP: Luminescent nanoprobe
ONOO^−^: Peroxynitrite
^1^O_2_: Singlet oxygen
O_2_˙^−^: Superoxide anion
PEG: Poly-ethylene glycol
pH: Scale used to specify the acidity or basicity of an aqueous solution
PDT: Photodynamic therapy
PS: Photosensitizer
PTT: Photothermal therapy
SBR: Signal-to-background radio
SPNs: Semiconducting polymer nanoparticles
TNF-α: Tumor necrosis factor α
TME: Tumor microenvironment
TAE: Transcatheter arterial embolization
TUNEL: Terminal deoxynucleotidyl transferase-mediated dUTP nick-end labeling
TPP: Tetraphenylporphyrin
US: Ultrasound
UV: Ultraviolet

## Data Availability

No data was collected during the preparation of this manuscript

## References

[cit1] James M. L., Gambhir S. S. (2012). A Molecular Imaging Primer: Modalities, Imaging Agents, and Applications. Physiol. Rev..

[cit2] Jiang Y., Pu K. (2021). Molecular Probes for Autofluorescence-Free Optical Imaging. Chem. Rev..

[cit3] Feng G., Zhang G.-Q., Ding D. (2020). Design of superior phototheranostic agents guided by Jablonski diagrams. Chem. Soc. Rev..

[cit4] Sun S.-K., Wang H.-F., Yan X.-P. (2018). Engineering Persistent Luminescence Nanoparticles for Biological Applications: From Biosensing/Bioimaging to Theranostics. Acc. Chem. Res..

[cit5] Shuhendler A. J., Pu K., Cui L., Uetrecht J. P., Rao J. (2014). Real-time imaging of oxidative and nitrosative stress in the liver of live animals for drug-toxicity testing. Nat. Biotechnol..

[cit6] Smith B. R., Gambhir S. S. (2017). Nanomaterials for *In Vivo* Imaging. Chem. Rev..

[cit7] Weissleder R., Pittet M. J. (2008). Imaging in the era of molecular oncology. Nature.

[cit8] Chen G., Yu J., Wu L., Ji X., Xu J., Wang C., Ma S., Miao Q., Wang L., Wang C., Lewis S. E., Yue Y., Sun Z., Liu Y., Tang B., James T. D. (2024). Fluorescent small molecule donors. Chem. Soc. Rev..

[cit9] He S., Song J., Qu J., Cheng Z. (2018). Crucial breakthrough of second near-infrared biological window fluorophores: design and synthesis toward multimodal imaging and theranostics. Chem. Soc. Rev..

[cit10] Li J., Pu K. (2019). Development of organic semiconducting materials for deep-tissue optical imaging, phototherapy and photoactivation. Chem. Soc. Rev..

[cit11] Cai Y., Wei Z., Song C., Tang C., Han W., Dong X. (2019). Optical nano-agents in the second near-infrared window for biomedical applications. Chem. Soc. Rev..

[cit12] Kenry, Duan Y., Liu B. (2018). Recent Advances of Optical Imaging in the Second Near-Infrared Window. Adv. Mater..

[cit13] Maldiney T., Bessière A., Seguin J., Teston E., Sharma S. K., Viana B., Bos A. J. J., Dorenbos P., Bessodes M., Gourier D., Scherman D., Richard C. (2014). The *in vivo* activation of persistent nanophosphors for optical imaging of vascularization, tumours and grafted cells. Nat. Mater..

[cit14] Shi T., Huang C., Li Y., Huang F., Yin S. (2022). NIR-II phototherapy agents with aggregation-induced emission characteristics for tumor imaging and therapy. Biomaterials.

[cit15] Du P., Wei Y., Liang Y., An R., Liu S., Lei P., Zhang H. (2023). Near-Infrared-Responsive Rare Earth Nanoparticles for Optical Imaging and Wireless Phototherapy. Adv. Sci..

[cit16] Hong G., Antaris A. L., Dai H. (2017). Near-infrared fluorophores for biomedical imaging. Nat. Biomed. Eng..

[cit17] Hong G., Lee J. C., Robinson J. T., Raaz U., Xie L., Huang N. F., Cooke J. P., Dai H. (2012). Multifunctional *in vivo* vascular imaging using near-infrared II fluorescence. Nat. Med..

[cit18] Li X., Yin C., Liew S. S., Lee C. S., Pu K. (2021). Organic Semiconducting Luminophores for Near-Infrared Afterglow, Chemiluminescence, and Bioluminescence Imaging. Adv. Funct. Mater..

[cit19] Yoon S., Kim M., Jang M., Choi Y., Choi W., Kang S., Choi W. (2020). Deep optical imaging within complex scattering media. Nat. Rev. Phys..

[cit20] Schmidt E. L., Ou Z., Ximendes E., Cui H., Keck C. H. C., Jaque D., Hong G. (2024). Near-infrared II fluorescence imaging. Nat. Rev. Methods Primers.

[cit21] Yan Y., Shi P., Song W., Bi S. (2019). Chemiluminescence and Bioluminescence Imaging for Biosensing and Therapy: *In Vitro* and *In Vivo* Perspectives. Theranostics.

[cit22] Vacher M., Galván I. F., Ding B.-W., Schramm S., Berraud-Pache R., Naumov P., Ferré N., Liu Y.-J., Navizet I., Roca-Sanjuán D., Baader W. J., Lindh R. (2018). Chemi- and Bioluminescence of Cyclic Peroxides. Chem. Rev..

[cit23] Gross S., Gammon S. T., Moss B. L., Rauch D., Harding J., Heinecke J. W., Ratner L., Piwnica-Worms D. (2009). Bioluminescence imaging of myeloperoxidase activity in vivo. Nat. Med..

[cit24] Zhan Z., Dai Y., Li Q., Lv Y. (2021). Small molecule-based bioluminescence and chemiluminescence probes for sensing and imaging of reactive species. Trends Anal. Chem..

[cit25] Wang X., Pu K. (2023). Molecular substrates for the construction of afterglow imaging probes in disease diagnosis and treatment. Chem. Soc. Rev..

[cit26] Wu Z., Midgley A. C., Kong D., Ding D. (2022). Organic persistent luminescence imaging for biomedical applications. Mater. Today Bio.

[cit27] Xu S., Chen R., Zheng C., Huang W. (2016). Excited State Modulation for Organic Afterglow: Materials and Applications. Adv. Mater..

[cit28] Qu R., Jiang X., Zhen X. (2024). Light/X-ray/ultrasound activated delayed photon emission of organic molecular probes for optical imaging: mechanisms, design strategies, and biomedical applications. Chem. Soc. Rev..

[cit29] Yang X., Waterhouse G. I. N., Lu S., Yu J. (2023). Recent advances in the design of afterglow materials: mechanisms, structural regulation strategies and applications. Chem. Soc. Rev..

[cit30] Xu C., Huang J., Jiang Y., He S., Zhang C., Pu K. (2022). Nanoparticles with ultrasound-induced afterglow luminescence for tumour-specific theranostics. Nat. Biomed. Eng..

[cit31] Wang Y., Yi Z., Guo J., Liao S., Li Z., Xu S., Yin B., Liu Y., Feng Y., Rong Q., Liu X., Song G., Zhang X.-B., Tan W. (2024). In vivo ultrasound-induced luminescence molecular imaging. Nat. Photon..

[cit32] Huang J., Su L., Xu C., Ge X., Zhang R., Song J., Pu K. (2023). Molecular radio afterglow probes for cancer radiodynamic theranostics. Nat. Mater..

[cit33] Xu C., Qin X., Wei X., Yu J., Zhang Y., Zhang Y., Ding D., Song J., Pu K. (2024). A cascade X-ray energy converting approach toward radio-afterglow cancer theranostics. Nat. Nanotechnol..

[cit34] Palner M., Pu K., Shao S., Rao J. (2015). Semiconducting Polymer Nanoparticles with Persistent Near-Infrared Luminescence for *In Vivo* Optical Imaging. Angew. Chem., Int. Ed..

[cit35] Miao Q., Xie C., Zhen X., Lyu Y., Duan H., Liu X., Jokerst J. V., Pu K. (2017). Molecular afterglow imaging with bright, biodegradable polymer nanoparticles. Nat. Biotechnol..

[cit36] Xie C., Zhen X., Miao Q., Lyu Y., Pu K. (2018). Self-Assembled Semiconducting Polymer Nanoparticles for Ultrasensitive Near-Infrared Afterglow Imaging of Metastatic Tumors. Adv. Mater..

[cit37] Cui D., Xie C., Li J., Lyu Y., Pu K. (2018). Semiconducting Photosensitizer-Incorporated Copolymers as Near-Infrared Afterglow Nanoagents for Tumor Imaging. Adv. Healthcare Mater..

[cit38] Qu R., He D., Wu M., Li H., Liu S., Jiang J., Wang X., Li R., Wang S., Jiang X., Zhen X. (2023). Afterglow/Photothermal Bifunctional Polymeric Nanoparticles for Precise Postbreast-Conserving Surgery Adjuvant Therapy and Early Recurrence Theranostic. Nano Lett..

[cit39] Jiang Y., Zhao M., Miao J., Chen W., Zhang Y., Miao M., Yang L., Li Q., Miao Q. (2024). Acidity-activatable upconversion afterglow luminescence cocktail nanoparticles for ultrasensitive *in vivo* imaging. Nat. Commun..

[cit40] He Y., Dong X., Zhao D., Han C., Jia Z., Meng H. M., Li Z. (2025). Exosome-Engineered Afterglow Probe for Targeted Imaging of Atherosclerotic Plaques In Vivo. Adv. Healthcare Mater..

[cit41] Zhao D., Zhou A., Zhang T., Han C., Meng H.-M., Lin Y., Li Z. (2025). Building high-contrast afterglow nanoprobe using semiconducting polymer nanoparticles and CuRu nanozyme for prolonged surgical navigation. Nano Today.

[cit42] Wang Y., Song G., Liao S., Qin Q., Zhao Y., Shi L., Guan K., Gong X., Wang P., Yin X., Chen Q., Zhang X. B. (2021). Cyclic Amplification of the Afterglow Luminescent Nanoreporter Enables the Prediction of Anti-cancer Efficiency. Angew. Chem., Int. Ed..

[cit43] Liao S., Wang Y., Li Z., Zhang Y., Yin X., Huan S., Zhang X.-B., Liu S., Song G. (2022). A novel afterglow nanoreporter for monitoring cancer therapy. Theranostics.

[cit44] Ni X., Zhang X., Duan X., Zheng H.-L., Xue X.-S., Ding D. (2018). Near-Infrared Afterglow Luminescent Aggregation-Induced Emission Dots with Ultrahigh Tumor-to-Liver Signal Ratio for Promoted Image-Guided Cancer Surgery. Nano Lett..

[cit45] Jiang Y., Huang J., Zhen X., Zeng Z., Li J., Xie C., Miao Q., Chen J., Chen P., Pu K. (2019). A generic approach towards afterglow luminescent nanoparticles for ultrasensitive *in vivo* imaging. Nat. Commun..

[cit46] Su X., Kong X., Sun K., Liu Q., Pei Y., Hu D., Xu M., Feng W., Li F. (2022). Enhanced Blue Afterglow through Molecular Fusion for Bio-applications. Angew. Chem., Int. Ed..

[cit47] Wang X., Yuan W., Xu M., Su X., Li F. (2021). Visualization of Acute Inflammation through a Macrophage-Camouflaged Afterglow Nanocomplex. ACS Appl. Mater. Interfaces.

[cit48] Zhang F., Xu M., Su X., Yuan W., Feng W., Su Q., Li F. (2021). Afterglow Implant for Arterial Embolization and Intraoperative Imaging. Chem.–Eur. J..

[cit49] Yang L., Zhao M., Chen W., Zhu J., Xu W., Li Q., Pu K., Miao Q. (2023). A Highly Bright Near-Infrared Afterglow Luminophore for Activatable Ultrasensitive *In Vivo* Imaging. Angew. Chem., Int. Ed..

[cit50] Zheng X., Wu W., Zheng Y., Ding Y., Xiang Y., Liu B., Tong A. (2021). Organic Nanoparticles with Persistent Luminescence for *In Vivo* Afterglow Imaging-Guided Photodynamic Therapy. Chem.–Eur. J..

[cit51] Anjong T. F., Choi H., Yoo J., Bak Y., Cho Y., Kim D., Lee S., Lee K., Kim B. G., Kim S. (2022). Multifunction-Harnessed Afterglow Nanosensor for Molecular Imaging of Acute Kidney Injury In Vivo. Small.

[cit52] Duan X., Zhang G. Q., Ji S., Zhang Y., Li J., Ou H., Gao Z., Feng G., Ding D. (2022). Activatable Persistent Luminescence from Porphyrin Derivatives and Supramolecular Probes with Imaging-Modality Transformable Characteristics for Improved Biological Applications. Angew. Chem., Int. Ed..

[cit53] Chen W., Zhang Y., Li Q., Jiang Y., Zhou H., Liu Y., Miao Q., Gao M. (2022). Near-Infrared Afterglow Luminescence of Chlorin Nanoparticles for Ultrasensitive *In Vivo* Imaging. J. Am. Chem. Soc..

[cit54] Liu Y., Teng L., Lou X.-F., Zhang X.-B., Song G. (2023). “Four-In-One” Design of a Hemicyanine-Based Modular Scaffold for High-Contrast Activatable Molecular Afterglow Imaging. J. Am. Chem. Soc..

[cit55] Lei L., Yang F., Meng X., Xu L., Liang P., Ma Y., Dong Z., Wang Y., Zhang X.-B., Song G. (2023). Noninvasive Imaging of Tumor Glycolysis and Chemotherapeutic Resistance *via De Novo* Design of Molecular Afterglow Scaffold. J. Am. Chem. Soc..

[cit56] Zhu J., Chen W., Yang L., Zhang Y., Cheng B., Gu W., Li Q., Miao Q. (2024). A Self-Sustaining Near-Infrared Afterglow Chemiluminophore for High-Contrast Activatable Imaging. Angew. Chem., Int. Ed..

[cit57] Han C., Jia Z., Wei C., Zhang T., Wang R., Meng H.-M., Li Z. (2025). A Novel Afterglow Molecular Probe for Monitoring of pH and Viscosity in Infected Wounds with Two-Dimensional Signal. Anal. Chem..

[cit58] Zhang Y., Xu W., Cheng D., Zhao M., Xiong J., Li Q., Miao Q. (2025). Molecular Engineering of a Self-Sustaining Modular Afterglow Scaffold for *In Vivo* Activatable Imaging. Angew. Chem., Int. Ed..

[cit59] Wang Y., Shen H., Li Z., Liao S., Yin B., Yue R., Guan G., Chen B., Song G. (2024). Enhancing Fractionated Cancer Therapy: A Triple-Anthracene Photosensitizer Unleashes Long-Persistent Photodynamic and Luminous
Efficacy. J. Am. Chem. Soc..

[cit60] Xu C., He S., Wei X., Huang J., Xu M., Pu K. (2023). Activatable Sonoafterglow Nanoprobes for T-Cell Imaging. Adv. Mater..

[cit61] Li J., Zhang G. Q., Zhang Y., Tang Y., Ding D., Li W., Liu Q. (2023). Building Highly Light-Harvesting Near-Infrared AIEgens Using Triazole-Based Luminescent Core for Improved Intravital Afterglow Imaging. Adv. Funct. Mater..

[cit62] He S., Xie C., Jiang Y., Pu K. (2019). An Organic Afterglow Protheranostic Nanoassembly. Adv. Mater..

[cit63] Chen C., Gao H., Ou H., Kwok R. T. K., Tang Y., Zheng D., Ding D. (2022). Amplification of Activated Near-Infrared Afterglow Luminescence by Introducing Twisted Molecular Geometry for Understanding Neutrophil-Involved Diseases. J. Am. Chem. Soc..

[cit64] Gao Z., Jia S., Ou H., Hong Y., Shan K., Kong X., Wang Z., Feng G., Ding D. (2022). An Activatable Near-Infrared Afterglow Theranostic Prodrug with Self-Sustainable Magnification Effect of Immunogenic Cell Death. Angew. Chem., Int. Ed..

[cit65] Lyu Y., Cui D., Huang J., Fan W., Miao Y., Pu K. (2019). Near-Infrared Afterglow Semiconducting Nano-Polycomplexes for the Multiplex Differentiation of Cancer Exosomes. Angew. Chem., Int. Ed..

[cit66] Wu L., Ishigaki Y., Hu Y., Sugimoto K., Zeng W., Harimoto T., Sun Y., He J., Suzuki T., Jiang X., Chen H.-Y., Ye D. (2020). H2S-activatable near-infrared afterglow luminescent probes for sensitive molecular imaging in vivo. Nat. Commun..

[cit67] Zeng W., Wu L., Ishigaki Y., Harimoto T., Hu Y., Sun Y., Wang Y., Suzuki T., Chen H. Y., Ye D. (2021). An Activatable Afterglow/MRI Bimodal Nanoprobe with Fast Response to H2S for *In Vivo* Imaging of Acute Hepatitis. Angew. Chem., Int. Ed..

[cit68] Banerjee J., Dutta K. (2021). A short overview on the synthesis, properties and major applications of poly(p-phenylene vinylene). Chem. Pap..

[cit69] Wang Y., Yin X.-B. (2025). Persistent luminescence materials for imaging and therapeutic applications. Coord. Chem. Rev..

[cit70] Han L., Liu J., Wei Z., Gu Y., Zhu Y., Chen L., Zhang X., Wu W., He F., Tian L. (2023). Structural Morphology Changes the Fate of Semiconducting Polymers in Afterglow Luminescence Imaging. Chem. Mater..

[cit71] Wu L., Sun Y., Sugimoto K., Luo Z., Ishigaki Y., Pu K., Suzuki T., Chen H.-Y., Ye D. (2018). Engineering of Electrochromic Materials as Activatable Probes for Molecular Imaging and Photodynamic Therapy. J. Am. Chem. Soc..

[cit72] Zhen X., Zhang C., Xie C., Miao Q., Lim K. L., Pu K. (2016). Intraparticle Energy Level Alignment of Semiconducting Polymer Nanoparticles to Amplify Chemiluminescence for Ultrasensitive *In Vivo* Imaging of Reactive Oxygen Species. ACS Nano.

[cit73] Lyu Y., Xie C., Chechetka S. A., Miyako E., Pu K. (2016). Semiconducting Polymer Nanobioconjugates for Targeted Photothermal Activation of Neurons. J. Am. Chem. Soc..

[cit74] Lu C., Zhang C., Wang P., Zhao Y., Yang Y., Wang Y., Yuan H., Qu S., Zhang X., Song G., Pu K. (2020). Light-free Generation of Singlet Oxygen through Manganese-Thiophene Nanosystems for pH-Responsive Chemiluminescence Imaging and Tumor Therapy. Chem.

[cit75] Hananya N., Eldar Boock A., Bauer C. R., Satchi-Fainaro R., Shabat D. (2016). Remarkable Enhancement of Chemiluminescent Signal by Dioxetane–Fluorophore Conjugates: Turn-ON Chemiluminescence Probes with Color Modulation for Sensing and Imaging. J. Am. Chem. Soc..

[cit76] Schaap A. P., Chen T.-S., Handley R. S., DeSilva R., Giri B. P. (1987). Chemical and enzymatic triggering of 1,2-dioxetanes. 2: fluoride-induced chemiluminescence from tert-butyldimethylsilyloxy-substituted dioxetanes. Tetrahedron Lett..

[cit77] Green O., Gnaim S., Blau R., Eldar-Boock A., Satchi-Fainaro R., Shabat D. (2017). Near-Infrared Dioxetane Luminophores with Direct Chemiluminescence Emission Mode. J. Am. Chem. Soc..

[cit78] Bruemmer K. J., Green O., Su T. A., Shabat D., Chang C. J. (2018). Chemiluminescent Probes for Activity-Based Sensing of Formaldehyde Released from Folate Degradation in Living Mice. Angew. Chem., Int. Ed..

[cit79] Hananya N., Green O., Blau R., Satchi-Fainaro R., Shabat D. (2017). Highly-Efficient Chemiluminescence Probe for the Detection of Singlet Oxygen in Living Cells. Angew. Chem., Int. Ed..

[cit80] Ullman E. F., Kirakossian H., Singh S., Wu Z. P., Irvin B. R., Pease J. S., Switchenko A. C., Irvine J. D., Dafforn A., Skold C. N., Wagner D. B. (1994). Luminescent oxygen channeling immunoassay: measurement of particle binding kinetics by chemiluminescence. Proc. Natl. Acad. Sci. U. S. A..

[cit81] Chan W.-L., Xie C., Lo W.-S., Bünzli J.-C. G., Wong W.-K., Wong K.-L. (2021). Lanthanide–tetrapyrrole complexes: synthesis, redox chemistry, photophysical properties, and photonic applications. Chem. Soc. Rev..

[cit82] Lo P., Leng X., Ng D. (2007). Hetero-arrays of porphyrins and phthalocyanines. Coord. Chem. Rev..

[cit83] Mellish K. J., Cox R. D., Vernon D. I., Griffiths J., Brown S. B. (2002). In Vitro Photodynamic Activity of a Series of Methylene Blue Analogues. Photochem. Photobiol..

[cit84] De Crozals G., Farre C., Sigaud M., Fortgang P., Sanglar C., Chaix C. (2015). Methylene blue phosphoramidite for DNA labelling. Chem. Commun..

[cit85] Farhadi A., Ho G. H., Sawyer D. P., Bourdeau R. W., Shapiro M. G. (2019). Ultrasound imaging of gene expression in mammalian cells. Science.

[cit86] Heiles B., Terwiel D., Maresca D. (2021). The Advent of Biomolecular Ultrasound Imaging. Neuroscience.

[cit87] Wei Y., Wang J. (2024). X-ray/γ-ray/Ultrasound-Activated Persistent Luminescence Phosphors for Deep Tissue Bioimaging and Therapy. ACS Appl. Mater. Interfaces.

[cit88] Lin X., Song J., Chen X., Yang H. (2020). Ultrasound-Activated Sensitizers and Applications. Angew. Chem., Int. Ed..

[cit89] Xu C., Huang J., Pu K. (2025). Sonoafterglow nanoprobes for deep-tissue imaging of peroxynitrite. Nat. Protoc..

[cit90] Huang K.-W., Chieh J.-J., Yeh C.-K., Liao S.-H., Lee Y.-Y., Hsiao P.-Y., Wei W.-C., Yang H.-C., Horng H.-E. (2017). Ultrasound-Induced Magnetic Imaging of Tumors Targeted by Biofunctional Magnetic Nanoparticles. ACS Nano.

[cit91] Li Z., Liu H., Zhang X.-B. (2024). Reactive oxygen species-mediated organic long-persistent luminophores light up biomedicine: from two-component separated nano-systems to integrated uni-luminophores. Chem. Soc. Rev..

[cit92] Xu C., Pu K. (2024). Illuminating cancer with sonoafterglow. Nat. Photon..

[cit93] Ou X., Qin X., Huang B., Zan J., Wu Q., Hong Z., Xie L., Bian H., Yi Z., Chen X., Wu Y., Song X., Li J., Chen Q., Yang H., Liu X. (2021). High-resolution X-ray luminescence extension imaging. Nature.

[cit94] Hong Z., Chen Z., Chen Q., Yang H. (2022). Advancing X-ray Luminescence for Imaging, Biosensing, and Theragnostics. Acc. Chem. Res..

[cit95] Withers P. J., Bouman C., Carmignato S., Cnudde V., Grimaldi D., Hagen C. K., Maire E., Manley M., Du Plessis A., Stock S. R. (2021). X-ray computed tomography. Nat. Rev. Methods Primers.

[cit96] Gan N., Zou X., Dong M., Wang Y., Wang X., Lv A., Song Z., Zhang Y., Gong W., Zhao Z., Wang Z., Zhou Z., Ma H., Liu X., Chen Q., Shi H., Yang H., Gu L., An Z., Huang W. (2022). Organic phosphorescent scintillation from copolymers by X-ray irradiation. Nat. Commun..

[cit97] Wang X., Shi H., Ma H., Ye W., Song L., Zan J., Yao X., Ou X., Yang G., Zhao Z., Singh M., Lin C., Wang H., Jia W., Wang Q., Zhi J., Dong C., Jiang X., Tang Y., Xie X., Yang Y., Wang J., Chen Q., Wang Y., Yang H., Zhang G., An Z., Liu X., Huang W. (2021). Organic phosphors with bright triplet excitons for efficient X-ray-excited luminescence. Nat. Photon..

[cit98] Xu C., Pu K. (2024). Organic radio-afterglow nanoprobes for cancer theranostics. Nat. Nanotechnol..

[cit99] Ma W., Su Y., Zhang Q., Deng C., Pasquali L., Zhu W., Tian Y., Ran P., Chen Z., Yang G., Liang G., Liu T., Zhu H., Huang P., Zhong H., Wang K., Peng S., Xia J., Liu H., Liu X., Yang Y. M. (2021). Thermally activated delayed fluorescence (TADF) organic molecules for efficient X-ray scintillation and imaging. Nat. Mater..

